# Toward smart agriculture: a hybrid mamba-transformer vision framework for plant disease detection

**DOI:** 10.3389/fpls.2026.1846242

**Published:** 2026-05-29

**Authors:** Qi Zhang, Junchao Zhao, Yuan-Li Cai, Kexin Liu, Yuheng Li

**Affiliations:** 1 Test Teaching and Research Office, Rocket Force University of Engineering, Xi’an, Shaanxi, China; 2 School of Automation Science and Engineering, Xi’an Jiaotong University, Xi’an, Shaanxi, China; 3 School of Information Engineering, Engineering University of PAP, Xi’an, Shaanxi, China; 4 Key Laboratory of Internet Information Retrieval of Hainan Province, Hainan University, Haikou, China

**Keywords:** hybrid deep learning, mamba architecture, plant disease detection, precision agriculture, vision transformer

## Abstract

Plant disease detection under complex field conditions remains a critical challenge for precision agriculture due to varying illumination, scale variations, subtle lesion patterns, and inter-class visual ambiguity. This study proposes MAFusionNet, a disease-aware hybrid vision framework integrating Mamba and Transformer architectures, with components explicitly designed for plant disease-specific challenges. The MAFusion Mixer operates parallel CS-Mamba and self-attention branches to simultaneously capture sequential lesion boundary evolution and global diseasecontext spatial relationships. The CS-Mamba branch employs the SS2D-LS Block with twodimensional selective scanning and Local-Selective enhancement for linear-complexity longrange modeling while preserving 2D lesion morphology. The PConv operator uses asymmetric directional kernels forming cross-shaped receptive fields to capture anisotropic disease patterns such as vein-aligned blights and directional rust streaks. We constructed PD40, a large-scale dataset with 80,369 expert-verified annotated images across 40 disease categories spanning eight major crops, with inter-annotator agreement Cohen’s *κ* = 0.874. Extensive experiments demonstrate that MAFusionNet achieves 94.7% mAP^50^ and 81.8% mAP^50:95^ on PD40, surpassing 25 state-of-the-art baselines including recent hybrid Mamba-Transformer detectors (CropMamba, HybridMamba, Mamba-DETR), with comprehensive ablation studies validating each component’s non-redundant contribution. Edge deployment analysis on NVIDIA Jetson hardware demonstrates practical feasibility: the compressed MAFusionNet-T-Lite variant (8.7M parameters) achieves 89.3% mAP^50^ at 18.4 FPS on Jetson Nano with 8.3W power consumption. The dataset and code are available at PD40-Dataset GitHub Repository.

## 1 Introduction

Plant diseases threaten global food security by reducing crop yield and quality substantially Savary et al. (2019). The Food and Agriculture Organization (FAO) reports that plant diseases cause annual crop losses of 20% to 40%, resulting in economic damages exceeding hundreds of billions of dollars Food and Agriculture Organization (2021). Recent studies further highlight the urgency of this challenge: Xie et al. (2025) demonstrated that climate change significantly threatens food security resilience in China, where the majority of the PD40 dataset was collected, underscoring the need for robust automated disease monitoring systems. From a biological perspective, Wang et al. (2026) showed that ERF transcription factors coordinate complex plant stress responses, revealing the molecular-level diversity of plant-pathogen interactions that manifests as the wide range of visual symptoms our detection system must recognize. Accurate and timely disease detection enables effective control measures, reduces pesticide usage, and supports sustainable agricultural production. Manual inspection by agricultural experts remains the traditional approach, but this method is time-consuming, labor-intensive, subjective, and unsuitable for large-scale monitoring Barbedo (2019).

Computer vision and deep learning have advanced automated plant disease detection significantly Mustofa et al. (2023); Ferentinos (2018). Object detection methods localize and classify disease regions simultaneously, making them valuable for precise monitoring and targeted treatment. CNN-based frameworks like YOLO Redmon et al. (2016) and Faster R-CNN Ren et al. (2015) are widely used due to their strong feature extraction and localization performance He et al. (2016); Krizhevsky et al. (2012). Yet CNNs have limitations: their local receptive fields constrain long-range spatial dependency modeling, reducing their effectiveness in capturing contextual information across large areas, essential for detecting multiple disease instances and distinguishing visually similar symptoms Mohanty et al. (2016).

Transformer-based detection architectures like DETR Carion et al. (2020) address CNN limitations by using self-attention mechanisms for global dependency modeling. DEIM Huang et al. (2024) accelerates DETR convergence with improved matching strategies. RT-DETR Lv et al. (2023) and its successors (RTDETRv2 Zhao et al. (2024), RT-DETRv3 Wang et al. (2024e)) apply Transformers to real-time detection, outperforming traditional CNN detectors Chen et al. (2023b); Thapa et al. (2023). However, self-attention’s quadratic complexity relative to sequence length creates deployment challenges in resource-constrained agricultural settings, especially on edge devices Shamshad et al. (2023).

State Space Models (SSMs), especially Mamba Gu and Dao (2023), offer an efficient alternative for sequence modeling with linear complexity. Mamba uses selective state-space mechanisms to capture long-range dependencies efficiently. Vision Mamba (VMamba) Liu et al. (2024) shows promising results in computer vision by incorporating two-dimensional selective scanning. Despite this progress, Mamba-based architectures remain underexplored for object detection, particularly in plant disease detection Zhang et al. (2024); Wang et al. (2024c).

Current detection approaches typically rely on single architectural paradigms, missing opportunities to combine complementary strengths across model families. YOLO and similar CNN detectors capture local spatial patterns well and run in real-time, but handle long-range dependencies poorly. DETR and other Transformer detectors model global context effectively but require substantial computation. Mamba-based methods provide efficient linear-complexity sequence modeling yet may miss multi-scale spatial features needed for accurate disease localization.

Real-world agricultural disease detection encounters several practical challenges. Field lighting varies considerably with time of day, weather, and canopy shadows, causing substantial appearance changes in captured images Barbedo (2016). Disease symptoms span multiple scales, from small isolated spots to large infection areas, requiring models that handle both fine and coarse patterns Arsenovic et al. (2019). Lesion patterns show subtle differences in color, texture, and morphology, demanding highly discriminative features Hughes and Salathé (2015). Agricultural settings have limited computational resources, requiring efficient architectures suitable for edge devices Kamilaris and Prenafeta-Boldú (2018).

We propose MAFusionNet, a hybrid vision detection framework that integrates Mamba and Transformer architectures for efficient plant disease detection. Unlike prior hybrid vision models that generically combine different architectural paradigms, MAFusionNet is explicitly motivated by three domain-specific challenges in plant disease detection: (1) *disease lesion anisotropy*—lesions such as rust streaks and veinaligned blights exhibit strong directional patterns requiring orientation-sensitive feature extraction rather than isotropic convolutions; (2) *cross-scale symptom co-occurrence*—a single leaf may simultaneously present tiny early-stage spots and large necrotic regions, necessitating joint modeling of local fine-grained textures and global structural context within a unified framework; (3) *inter-class visual ambiguity* (i.e., visual similarity *between* different disease categories)—diseases like early blight and septoria leaf spot share similar color and texture profiles, demanding highly discriminative features that capture both sequential texture evolution along lesion boundaries and holistic spatial relationships. These challenges cannot be adequately addressed by any single architectural paradigm: CNNs miss long-range context needed for ambiguity resolution, Transformers lack efficiency for dense multi-scale processing, and pure Mamba models lose 2D spatial coherence during sequence flattening. MAFusionNet resolves these through targeted component designs that synergistically address each challenge.

This work makes four main contributions:

- Disease-Aware Hybrid Architecture: MAFusionNet introduces MAFusion Mixer modules that operate CS-Mamba and self-attention branches in parallel with learned content-dependent fusion. This design is specifically motivated by the need to simultaneously capture sequential lesion boundary evolution (via Mamba’s causal state propagation) and global disease-healthy tissue relationships (via attention’s pairwise associations), enabling disambiguation of visually similar diseases that require both local texture discrimination and holistic spatial reasoning.
- Lesion-Oriented Structural Components: The SS2D-LS Block extends selective scanning to 2D with a Local-Selective enhancement stage that establishes spatial coherence before global state propagation, directly addressing the loss of 2D lesion morphology information in naive 1D scanning. The PConv operator employs asymmetric directional kernels forming cross-shaped receptive fields, explicitly designed to capture anisotropic disease patterns such as elongated rust streaks along leaf veins and directional blight progression—patterns that isotropic square convolutions dilute with redundant diagonal computation.
- Large-Scale Annotated Benchmark with Quality Assurance: PD40 dataset contains 80,369 images with expert-verified bounding box annotations across 40 disease categories spanning eight major crops. The dataset was constructed with rigorous quality control including dual-expert annotation verification with inter-annotator agreement metrics, geographic diversity across multiple climate zones, and multi-device image acquisition to ensure robustness.
- Comprehensive Evaluation with Practical Validation: Extensive experiments demonstrate stateof-the-art detection performance compared to 25 baselines including recent hybrid detectors, with thorough ablation studies validating each component’s non-redundant contribution. We additionally provide edge deployment analysis on resource-constrained hardware, cross-dataset generalization evaluation, and detailed error analysis with per-class confusion quantification to validate practical applicability.

The remainder of this paper is organized as follows: Section 2 reviews related work on plant disease identification and relevant deep learning architectures. Section 3 presents the detailed methodology of MAFusionNet, including the MAFusion Mixer, CS-Mamba, SS2D-LS Block, and PConv operator. Section 4 describes the construction of the PD40 dataset. Section 5 presents experimental setup, results, and comprehensive analyses. Section 6 concludes the paper with a discussion of future directions.

## 2 Related work

### 2.1 Object detection approaches for plant disease detection

Deep learning has transformed plant disease identification over the past decade. Early works focused on classification, but recent research increasingly uses object detection frameworks for precise disease region localization. Object detection provides spatial localization through bounding boxes, enabling accurate disease assessment, targeted treatment planning, and multi-disease detection in single images, capabilities essential for precision agriculture.

CNN-based Detectors: YOLO (You Only Look Once) Redmon et al. (2016) dominates real-time object detection. Its single-stage approach achieves fast inference by predicting bounding boxes and class probabilities directly from full images in one forward pass. Recent YOLO variants (YOLOv5-v13) advance plant disease detection through cross-stage partial networks, path aggregation networks, and anchor-free detection heads Raza et al. (2025). Alazeb et al. (2024) demonstrated the potential of remote intelligent perception systems for multi-object detection in real-world monitoring scenarios, further validating the applicability of real-time detection frameworks to agricultural contexts. Zhu et al. (2023) proposed Apple-Net, an improved YOLOv5 model for apple leaf disease detection, achieving 95.9% mAP50 across multiple disease categories. Two-stage detectors like Faster R-CNN Ren et al. (2015) use region proposal networks followed by refinement, typically achieving higher localization accuracy but requiring more computation. Jiang et al. showed Faster R-CNN’s effectiveness for apple leaf disease detection in complex field conditions, achieving 78.6% mAP^50^ with multi-scale feature pyramids.

Transformer-based Detectors: DETR Carion et al. (2020) introduced end-to-end detection via bipartite matching and Transformers, removing hand-designed components like anchor generation and non-maximum suppression. Yet DETR’s quadratic attention complexity and slow convergence hinder agricultural deployment. DEIM Huang et al. (2024) addresses this with dense one-to-one matching and matchability-aware loss, cutting training time by 50% while maintaining accuracy. RT-DETR Lv et al. (2023) uses efficient encoders and IoU-aware query selection for competitive accuracy with faster inference. RT-DETRv2 Zhao et al. (2024) adds bag-of-freebies techniques, while RT-DETRv3 Wang et al. (2024e) introduces hierarchical dense positive supervision with CNN auxiliary branches and self-attention perturbation, achieving state-of-the-art real-time detection on COCO.

Existing detectors face challenges for plant disease detection. CNN detectors extract local patterns well but struggle with global context needed to distinguish subtle disease variations. Transformer detectors capture comprehensive dependencies but require prohibitive computation. These limitations motivate hybrid architectures balancing accuracy, efficiency, and global modeling.

### 2.2 Vision transformers and attention mechanisms

The introduction of Vision Transformers (ViTs) Dosovitskiy et al. (2020) has fundamentally transformed computer vision by demonstrating that pure attention-based architectures can match or exceed CNNs in image recognition tasks. By treating images as sequences of patches and applying self-attention mechanisms, ViTs effectively capture global dependencies and long-range relationships that CNNs struggle to model with their inherently local receptive fields. Several recent studies have successfully adapted Transformer architectures for plant disease identification, achieving notable improvements over traditional CNN approaches. Chen et al. (2023b) proposed a hybrid CNN-Transformer architecture that synergistically combines local feature extraction from convolutional layers with global dependency modeling from Transformer blocks, demonstrating that this complementary fusion yields superior performance for disease recognition. Thapa et al. (2023) introduced PlantViT, a pure Transformer-based model specifically designed for plant disease detection, showing that self-attention mechanisms excel at capturing subtle disease patterns and inter-class discriminative features across multiple plant disease datasets.

Hierarchical vision Transformers, particularly Swin Transformer Liu et al. (2021b), have proven especially effective for dense prediction tasks through their efficient shifted window mechanism and multi-scale feature representation. Zhou et al. (2024) showed that channel-wise attention mechanisms in Transformer architectures enable fine-grained feature discrimination, supporting the broader trend of attention-based architectures for detailed visual analysis. Several works have adapted Swin Transformer for plant disease detection Liu et al. (2021a); Yu et al. (2023), achieving state-of-the-art results while maintaining more reasonable computational costs compared to vanilla ViTs. Khan et al. (2025) proposed a dense-inception architecture with attention modules for plant disease classification, demonstrating that attention-enhanced networks significantly improve fine-grained recognition of disease symptoms across diverse plant species. The recently proposed Plant-XViT Agarwal et al. (2024) combines CNNs with Vision Transformers in a lightweight framework specifically optimized for efficient plant disease identification across multiple crops, demonstrating that carefully designed hybrid architectures can achieve excellent accuracy with reduced parameters and computational requirements.

Despite these impressive advances, Transformer-based approaches still face significant challenges in practical agricultural deployment scenarios. The quadratic computational complexity of self-attention with respect to input sequence length results in substantial memory consumption and inference latency, particularly for high-resolution agricultural images. This computational burden poses serious obstacles for deployment on resource-constrained edge devices commonly used in agricultural monitoring systems. Furthermore, Transformers typically require very large training datasets to learn effective representations, which may not always be available for emerging diseases or underrepresented crop species. Self-supervised learning approaches, such as the method proposed by Tian et al. (2024) for learning effective representations from limited labeled data, offer a potential direction for addressing this data scarcity challenge. These limitations motivate exploration of alternative architectures that can maintain global modeling capabilities while significantly reducing computational overhead, making them more suitable for practical agricultural applications.

### 2.3 State space models and hybrid architectures

State Space Models (SSMs) have recently emerged as a highly promising alternative for efficient sequence modeling with linear computational complexity. The Structured State Space Sequence (S4) model Gu et al. (2021) introduced structured parameterization enabling efficient computation while maintaining impressive long-range modeling capabilities. Building upon S4, the Mamba architecture Gu and Dao (2023) incorporates selective state-space mechanisms that allow the model to dynamically focus on relevant information while filtering out irrelevant content, achieving remarkable performance across various sequence modeling tasks. The selective scanning mechanism in Mamba enables linear-complexity modeling of long-range dependencies, making it particularly attractive for resource-constrained applications where computational efficiency is paramount.

Recent works have begun exploring Mamba’s potential for computer vision tasks. Vision Mamba (VMamba) Liu et al. (2024) adapts Mamba for image classification by flattening 2D feature maps into 1D sequences and applying bidirectional selective scanning, demonstrating competitive performance with reduced computational costs compared to Transformer-based models. U-Mamba Ma et al. (2024) extends this approach to medical image segmentation, showing that Mamba-based architectures can effectively capture spatial dependencies with superior efficiency. For plant disease identification specifically, PlantMamba Zhang et al. (2024) applies Vision Mamba to plant leaf disease classification, demonstrating competitive performance with CNN and Transformer baselines while requiring fewer computational resources. ConMamba Wang et al. (2024d) integrates a Vision Mamba encoder with contrastive learning mechanisms to improve feature discrimination for plant disease recognition.

The concept of hybrid architectures that strategically combine strengths of different model families has gained increasing attention in recent vision research. CoAtNet Dai et al. (2021) combines convolution and self-attention in a unified framework through careful co-design of convolutional and attention blocks, achieving strong performance on large-scale image recognition. The multi-encoder design philosophy has also proven effective in other visual computing domains; for instance, Luo et al. (2025) demonstrated that parallel multi-encoder architectures based on conditional diffusion models can achieve robust performance for image watermarking, validating the general effectiveness of multi-branch parallel processing strategies. ConvNeXt Liu et al. (2022) revisits pure ConvNets with modern training techniques and architectural innovations inspired by Transformers, demonstrating that carefully modernized CNNs can match Transformer performance. For plant disease identification, several multi-branch architectures have been explored: DualNet Wang et al. (2021) employs dual branches for multi-scale feature extraction, while HybridNet Zhang et al. (2022) combines CNN and LSTM for temporal modeling of disease progression. FasterNet Chen et al. (2023a) introduces a systematic approach to partial convolution for efficient feature extraction, demonstrating strong performance-efficiency trade-offs. Inspired by this work, we design a specialized PConv operator with “cross-shaped” receptive field expansion tailored for preserving fine disease lesion textures and structural edges, which is critical for accurate plant disease identification.

More recently, hybrid Mamba-Transformer architectures have emerged as a promising research direction. Mamba-DETR Li et al. (2024) replaces the Transformer encoder in DETR with Mamba blocks for efficient detection, achieving competitive accuracy with reduced computational cost on general object detection benchmarks. HybridMamba Zhao et al. (2025) proposes a unified backbone that interleaves Mamba and Transformer layers for visual recognition tasks. In the agricultural domain, AgriVision-Mamba Chen et al. (2025) applies hybrid state space models to agricultural object detection, and CropMamba Wang et al. (2025) combines Mamba with Transformer for crop disease detection in field environments. PlantDet-Mamba Huang et al. (2025) introduces multi-scale Mamba-based detection for plant diseases. MambaFusion Xu et al. (2024) explores efficient multi-scale feature fusion via selective state spaces, and EfficientVMamba Zhang et al. (2025) proposes atrous selective scanning for lightweight visual Mamba architectures.

However, these existing hybrid approaches primarily focus on *generic* architectural combination strategies—interleaving or stacking Mamba and Transformer layers—without designing components that specifically target the unique visual characteristics of plant disease patterns. Our work differs fundamentally in three aspects: (1) the MAFusion Mixer’s parallel fusion is explicitly motivated by the need to simultaneously capture sequential lesion boundary evolution and global disease-tissue spatial relationships; (2) the SS2D-LS Block incorporates a Local-Selective enhancement stage that preserves 2D lesion morphology information before global state propagation, addressing spatial coherence loss in standard 1D scanning; (3) the PConv operator’s cross-shaped receptive fields are specifically designed for anisotropic disease patterns such as vein-aligned blights and directional rust streaks. This domain-aware design philosophy distinguishes MAFusionNet from prior hybrid architectures that apply generic fusion strategies without considering disease-specific visual characteristics.

### 2.4 Datasets for plant disease detection

Dataset availability and quality directly impact plant disease detection system development. Early datasets focused on classification under controlled laboratory conditions, while recent work increasingly addresses object detection in real field environments where precise disease localization matters for practical deployment.

PlantVillage Hughes and Salathé (2015) established a foundation with 54,306 images across 38 disease categories, though its controlled laboratory setting limits field applicability. The Plant Pathology Challenge Thapa et al. (2020) provides high-resolution apple disease images with severity annotations. Various crop-specific datasets exist for tomato Brahimi et al. (2017); Fuentes et al. (2017); Liu and Wang (2020), rice Ma et al. (2017); Chen et al. (2021), and apple diseases Jiang et al. (2019), primarily targeting classification tasks.

For object detection, PlantDoc Singh et al. (2020) offers 2,598 field images with bounding boxes across 13 plant species and 17 disease classes, representing progress toward real-world scenarios but remaining limited in scale. IP102 Wu et al. (2019) demonstrates large-scale feasibility with 75,222 images for insect pest recognition across 102 categories. Data augmentation techniques help address scarcity, from traditional methods (rotation, flipping, color adjustment) Wang et al. (2017); Picon et al. (2019) to GANbased synthesis like LeafGAN Cap et al. (2020), though synthetic data struggles to fully replicate field complexity.

Despite these efforts, existing datasets face limitations including small scale (typically under 20,000 images), single-crop focus, controlled imaging conditions, classification-only labels, and limited disease coverage (rarely exceeding 30 categories). Our PD40 dataset addresses these gaps with 80,369 annotated images across 40 disease categories spanning eight major crops (strawberry, tomato, peanut, rice, potato, rubber tree, wheat, corn), providing detection-oriented bounding box annotations captured under diverse field conditions with varying illumination and complex backgrounds, making it a comprehensive resource for plant disease detection research and practical deployment.

**Figure 1:**
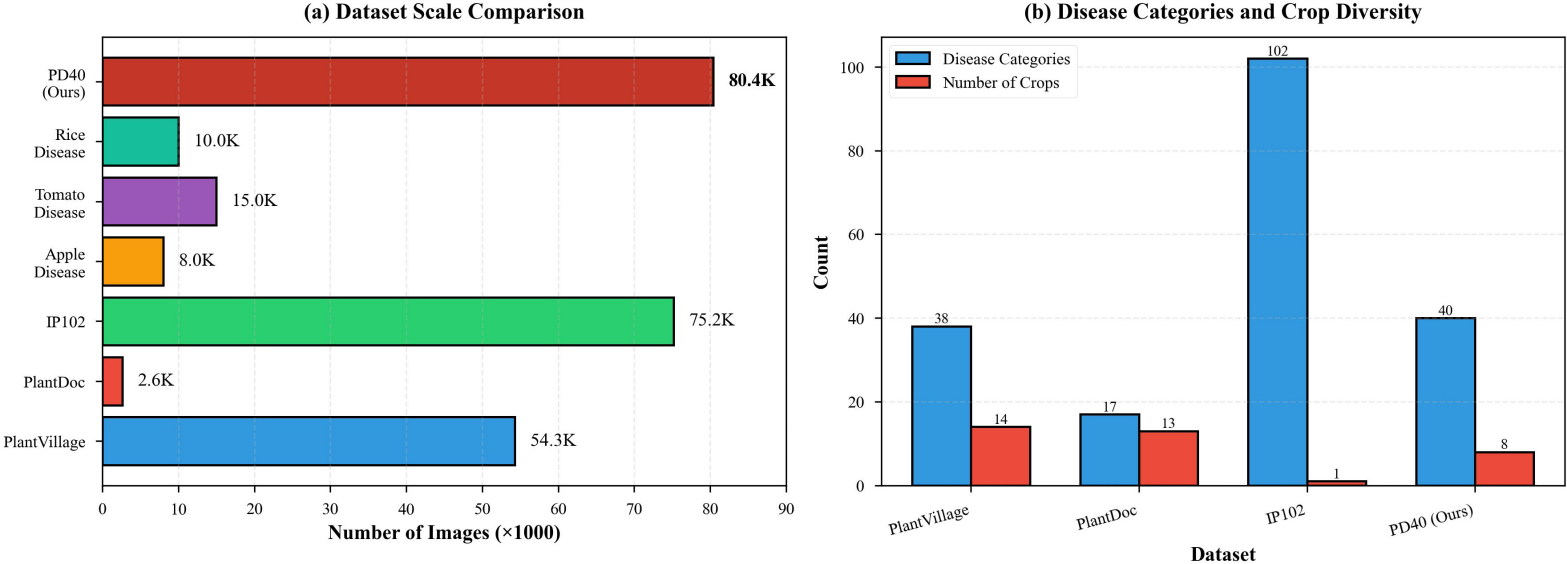
Quantitative comparison of plant disease datasets. **(a)** Dataset scale comparison showing PD40’s advantage in number of images. **(b)** Disease categories and crop diversity comparison, demonstrating PD40’s balanced coverage across multiple dimensions.

**Figure 1** provides a quantitative comparison of PD40 with existing plant disease datasets. As shown in **Figure 1a**, PD40 achieves the largest scale among detection-oriented datasets with 80.4K images, surpassing PlantDoc by 30× and comparable to IP102 while focusing on plant diseases rather than insect pests. **Figure 1b** demonstrates PD40’s balanced approach: while IP102 contains more categories (102), it focuses solely on insect pests; PD40 provides 40 disease categories across 8 diverse crops, offering broader agricultural applicability than single-crop datasets like PlantVillage (14 crops but classification-only) or PlantDoc (13 crops but limited scale).

## 3 Methodology

### 3.1 Architecture of MAFusionNet

Effective plant disease identification architectures must balance competing objectives: capturing finegrained local textures for discriminating subtle symptoms, establishing long-range spatial dependencies for understanding disease context, maintaining computational efficiency for resource-constrained deployment, and generalizing across diverse imaging conditions. Single-paradigm approaches face inherent trade-offs. CNNs extract local patterns well but lack global context. Transformers capture global dependencies but require excessive computation. Pure Mamba models achieve linear complexity but may miss multi-scale spatial features.

**Figure 2:**
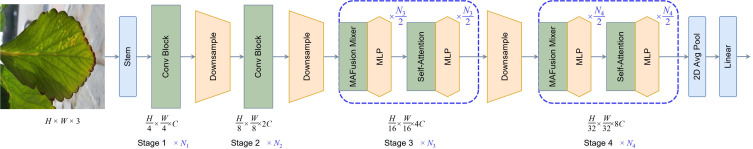
Architecture of MAFusionNet. The network employs a four-stage hierarchical design: Stages 1–2 use convolutional blocks for low-level feature extraction, while Stages 3–4 incorporate MAFusion Mixer modules for high-level semantic modeling with global context awareness.

MAFusionNet addresses these limitations through a hybrid architecture integrating convolutional operations, selective state-space mechanisms, and self-attention via hierarchical multi-stage design. The key insight: different semantic levels need different processing. Low-level features benefit from local convolutions preserving spatial structure and extracting texture primitives. High-level features require global receptive fields and cross-scale modeling for contextual relationships and abstract disease patterns. Strategic operator deployment at appropriate stages yields superior performance with reasonable computation. **Figure 2** shows the four-stage architecture with progressively increasing semantic abstraction and decreasing spatial resolution.

#### 3.1.1 Hierarchical multi-stage design philosophy

MAFusionNet uses a four-stage encoder progressively transforming raw pixels into semantically rich representations for disease detection. Each stage operates at different spatial scales with operators matching its semantic level and computational constraints, creating an efficient pipeline balancing accuracy, efficiency, and robustness.

#### 3.1.2 Stem layer and initial feature extraction

Given input RGB image $\text{X}\;\in \;{\mathbb{R}}^{H\times W\times 3}$, the network starts with a stem module performing spatial downsampling while expanding channel dimensionality for rich initial features. The stem transformation composes convolutional operations with progressive spatial reduction (Equation 1):

(1) $${\text{F}}_{0}=\phi_{\text{stem}}(\text{X};{\text{Θ}}_{\text{stem}})=\sigma (W_{2}*\sigma (W_{1}*\text{X}))\in {\mathbb{R}}^{\frac{H}{4}\times \frac{W}{4}\times C_{1}}$$

where $W_{1}$ and $W_{2}$ represent learnable convolutional kernels with stride 2, ∗ denotes the convolution operation, *σ*(·) represents the activation function (GELU), and ${\text{Θ}}_{\text{stem}}$ collectively denotes all learnable parameters in the stem. This 4× spatial reduction balances the trade-off between retaining fine-grained spatial details and reducing computational burden for subsequent stages. The expansion to *C*_1_ channels provides sufficient representational capacity for encoding low-level visual primitives including edges, corners, color gradients, and basic texture patterns that serve as building blocks for higher-level disease feature learning.

#### 3.1.3 Stage 1 and stage 2: local feature hierarchy construction

The first two stages focus exclusively on constructing hierarchical representations of local spatial patterns through purely convolutional operations. Each stage consists of multiple residual blocks with depth-wise separable convolutions and inverted bottleneck structures, formulated in Equation 2:

(2) $$\begin{array}{ll} {\text{F}}_{1} & =\phi_{\text{conv}}^{\left(1\right)}({\text{F}}_{0};{\text{Θ}}_{1})=\sum_{i=1}^{N_{1}}R_{i}^{\left(1\right)}(\cdot )\circ {\text{F}}_{0}\in {\mathbb{R}}^{\frac{H}{4}\times \frac{W}{4}\times C_{1}}\\ {{\text{F}}^{\prime }}_{1} & =D_{1}({\text{F}}_{1})=W_{\text{down}}^{\left(1\right)}*{\text{F}}_{1}\in {\mathbb{R}}^{\frac{H}{8}\times \frac{W}{8}\times C_{2}}\\ {\text{F}}_{2} & =\phi_{\text{conv}}^{\left(2\right)}({\text{F}}_{1}^{'};{\text{Θ}}_{2})=\sum_{i=1}^{N_{2}}R_{i}^{\left(2\right)}(\cdot )\circ {\text{F}}_{1}^{'}\in {\mathbb{R}}^{\frac{H}{8}\times \frac{W}{8}\times C_{2}} \end{array}$$

where $R_{i}^{\left(s\right)}$ denotes the *i*-th residual block in stage *s*, ° represents function composition, *N*_1_ and *N*_2_ are the numbers of blocks in each stage, and $D_{s}$ represents the downsampling operation with learnable parameters. Each residual block $R_{i}$ implements the transformation (Equation 3):

(3) $$R_{i}(\text{F})=\text{F}+\text{PWConv}(\text{DWConv}(\text{PWConv}(\text{LN}(\text{F}))))$$

where PWConv and DWConv denote pointwise and depthwise convolutions respectively, and LN represents layer normalization. This inverted bottleneck structure expands channels, applies spatial filtering, and projects back to the original dimension, enabling efficient extraction of multi-scale local patterns including fine lesion textures, color variations, and structural edges while maintaining linear computational growth relative to spatial dimensions.

#### 3.1.4 Stage 3 and stage 4: global context and cross-scale integration

The latter two stages introduce our proposed MAFusion Mixer modules to establish global dependencies and model cross-scale state representations. The forward propagation through these stages can be formulated as (Equation 4):

(4) $$\begin{array}{ll} {\text{F}}_{2}^{'} & =D_{2}({\text{F}}_{2})\in {\mathbb{R}}^{\frac{H}{16}\times \frac{W}{16}\times C_{3}}\\ {\text{F}}_{3} & ={\text{Φ}}_{\text{hybrid}}^{\left(3\right)}({{\text{F}}^{\prime }}_{2};{\text{Θ}}_{3})=\sum_{i=1}^{N_{3}}[\text{SA}(\text{MLP}(M_{i}({\text{F}}_{2}^{'})+{\text{F}}_{2}^{'}))+M_{i}(F_{2}^{'})]\\ {\text{F}}_{3}^{'} & =D_{3}({\text{F}}_{3})\in {\mathbb{R}}^{\frac{H}{32}\times \frac{W}{32}\times C_{4}}\\ {\text{F}}_{4} & ={\text{Φ}}_{\text{hybrid}}^{\left(4\right)}({\text{F}}_{3}^{'};{\text{Θ}}_{4})=\sum_{i=1}^{N_{4}}[\text{SA}(\text{MLP}(M_{i}(F_{3}^{'})+F_{3}^{'}))+M_{i}({F^{\prime }}_{3})] \end{array}$$

where $M_{i}$ denotes the *i*-th MAFusion Mixer block that integrates parallel CS-Mamba and self-attention branches (detailed in Section 3.2), SA represents the self-attention mechanism for additional global context refinement, MLP denotes multi-layer perceptron feedforward networks, and *N*_3_, *N*_4_ are the numbers of blocks in stages 3 and 4 respectively. The nested residual connections at both the block level and sub-block level facilitate gradient flow through the deep network and enable effective learning of residual transformations. By operating at reduced spatial resolutions ( $\frac{H}{16}\times \frac{W}{16}$ and $\frac{H}{32}\times \frac{W}{32}$), the quadratic complexity of self-attention becomes computationally feasible while still providing sufficient spatial granularity for capturing disease patterns across the entire leaf structure.

#### 3.1.5 Detection head and loss function

The multi-scale features from stages 2, 3, and 4 are fed into a detection head for object localization and classification. Following modern detection frameworks, we employ a Feature Pyramid Network (FPN) structure to aggregate multi-scale features (Equation 5):

(5) $${\text{P}}_{2},{\text{P}}_{3},{\text{P}}_{4}=\text{FPN}({\text{F}}_{2},{\text{F}}_{3},{\text{F}}_{4})$$

where ${\text{P}}_{i}$ denotes the feature pyramid at scale *i*. For each pyramid level, the detection head predicts bounding boxes, objectness scores, and class probabilities. The detection loss combines localization loss $L_{\text{box}}$, classification loss $L_{\text{cls}}$, and objectness loss $L_{\text{obj}}$ (Equation 6):

(6) $$L_{\text{total}}=\lambda_{\text{box}}L_{\text{box}}+\lambda_{\text{cls}}L_{\text{cls}}+\lambda_{\text{obj}}L_{\text{obj}}$$

where *λ*_box_, *λ*_cls_, *λ*_obj_ are loss weights. The box loss employs CIoU (Complete Intersection over Union) for accurate localization, the classification loss uses focal loss to handle class imbalance, and the objectness loss employs binary cross-entropy. This hierarchical architecture establishes an effective information processing pipeline: early convolutional stages build local feature hierarchies with spatial precision, middle hybrid stages establish global context and cross-scale dependencies, and the multi-scale detection head accurately localizes and classifies disease regions, achieving superior accuracy-efficiency balance for practical plant disease detection.

### 3.2 MAFusion mixer

**Figure 3:**
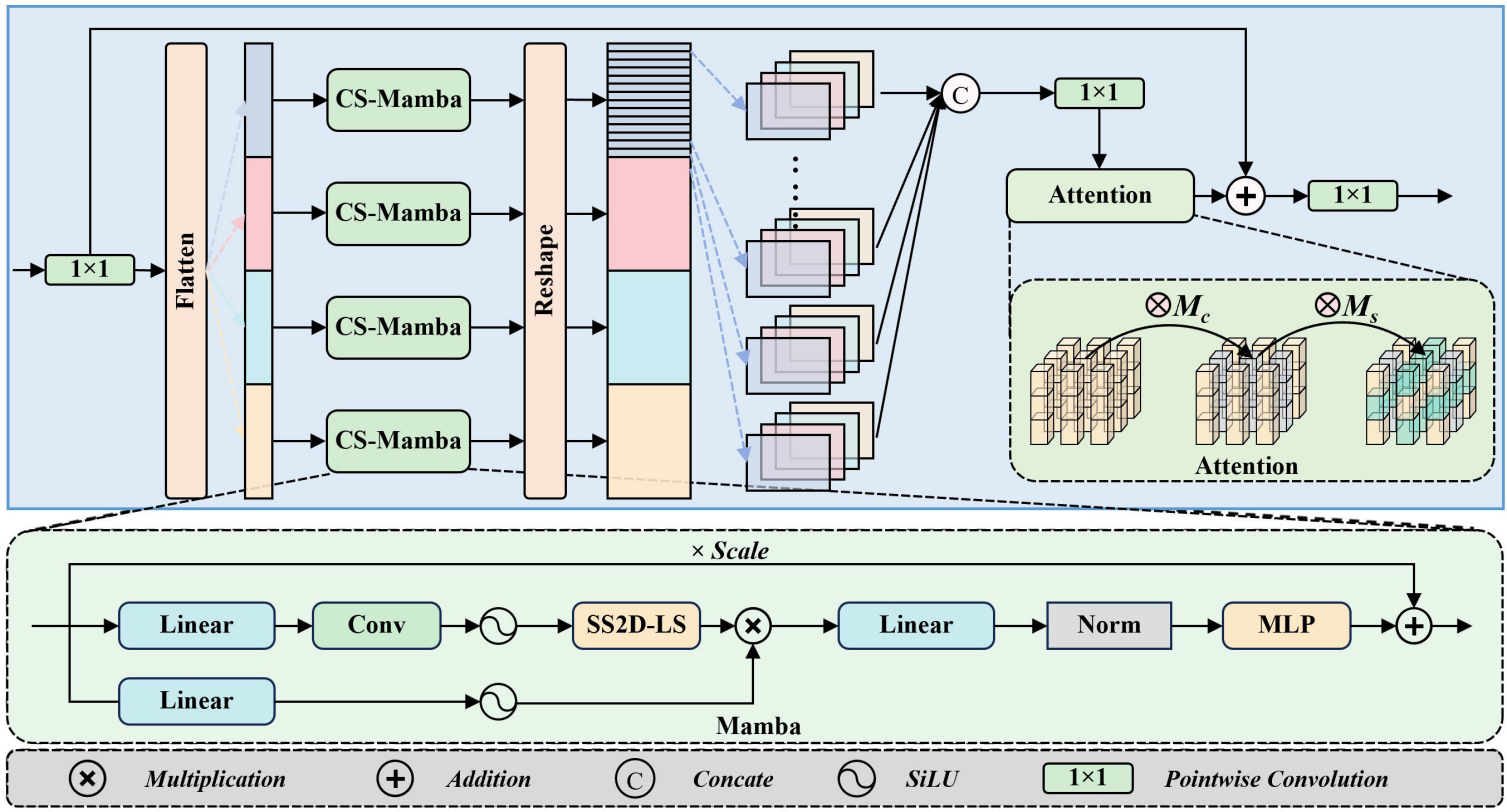
Detailed structure of MAFusion Mixer. The module consists of parallel CS-Mamba branches for efficient cross-scale state modeling and a self-attention branch for global context capture. The bottom panel shows the internal structure of the Mamba block with SS2D-LS as its core operator.

The MAFusion Mixer constitutes the core architectural innovation of MAFusionNet, designed to address a fundamental challenge specific to plant disease detection: the simultaneous need for *sequential lesion boundary modeling* and *global disease-context reasoning*. Disease lesions develop through progressive tissue degradation that creates characteristic texture gradients along lesion boundaries—from healthy green tissue through chlorotic yellowing to necrotic brown centers. This sequential evolution is naturally modeled by Mamba’s causal state propagation, which tracks how disease features evolve spatially across the lesion boundary. Simultaneously, distinguishing visually similar diseases (e.g., early blight vs. septoria leaf spot, which share similar brown spot morphology but differ in spatial distribution patterns relative to leaf veins and margins) requires global context that relates disease regions to the overall leaf structure—a capability provided by self-attention’s pairwise associations across all spatial positions. Neither mechanism alone suffices: Mamba misses non-local structural relationships, while self-attention cannot efficiently model the directional texture evolution along lesion gradients. By operating these mechanisms in parallel and fusing their outputs through learned content-dependent weighting, the MAFusion Mixer enables the model to dynamically emphasize sequential texture modeling for lesion boundary delineation and global spatial reasoning for inter-class disambiguation, depending on the input content (see **Figure 3**). 

#### 3.2.1 Input projection and channel splitting

Given input features $\text{X}\in {\mathbb{R}}^{H\times W\times C}$, the MAFusion Mixer applies normalization and projection (Equation 7):

(7) $${\text{X}}^{\prime }=\text{Flatten}(\phi_{\text{proj}}(\text{LN}(\text{X})))\in {\mathbb{R}}^{N\times C^{\prime }}$$

where LN(·) denotes layer normalization, $\phi_{\text{proj}}$ is a 1×1 convolution with expansion factor α=2 yielding $C^{\prime }=2C$ channels, and N=HW represents the flattened spatial sequence length.

#### 3.2.2 CS-mamba branch: multi-scale state evolution

The CS-Mamba branch implements selective state-space modeling through continuous-time dynamics. For input sequence **x**(*t*), the state evolution follows (Equation 8):

(8) $$\frac{d\text{h}(t)}{dt}=\text{A}(x)h(t)+\text{B}(x)x(t),\text{ y}(t)=\text{C}(x)\text{h}(t)$$

where $\text{h}(t)\in {\mathbb{R}}^{D_{h}}$ represents the latent state encoding temporal dependencies, and the state-space matrices $\text{A}(\text{x})\in {\mathbb{R}}^{D_{h}\times D_{h}}$, $\text{B}(\text{x})\in {\mathbb{R}}^{D_{h}\times D}$, $\text{C}(\text{x})\in {\mathbb{R}}^{D\times D_{h}}$ are input-dependent, enabling selective information propagation. The projected features are split into K=4 parallel groups, each maintaining independent state trajectories to capture cross-scale dependencies at different granularities, with outputs concatenated to form ${\text{Z}}_{\text{mamba}}\in {\mathbb{R}}^{H\times W\times C^{\prime }}$^′^.

#### 3.2.3 Self-attention branch: global association modeling

In parallel with the CS-Mamba pathway, a self-attention branch establishes comprehensive global dependencies through similarity-based pairwise interactions. The multi-head self-attention mechanism computes (Equation 9):

(9) $$Z_{\text{attn}}=\text{MultiHead}({\text{X}}^{\prime })=\text{Concat}({\text{head}}_{1},\ldots ,{\text{head}}_{h}){\text{W}}_{O}$$

where each attention head ${\text{head}}_{i}=\text{Attention}({\text{X}}^{\prime }{\text{W}}_{Q}^{i},{\text{X}}^{\prime }{\text{W}}_{K}^{i},{\text{X}}^{\prime }{\text{W}}_{V}^{i})$ computes scaled dot-product attention (Equation 10):

(10) $$\text{Attention}(\text{Q},\text{K},\text{V})=\text{Softmax}(\frac{\text{Q}{\text{K}}^{T}}{\sqrt{d_{k}}})\text{V}$$

with $d_{k}=C^{\prime }/h$ as the per-head dimension. This mechanism enables each spatial position to aggregate information from all other positions weighted by learned semantic similarity, capturing long-range dependencies with $O(N^{2})$ complexity.

#### 3.2.4 Complementary fusion: synergistic integration

The CS-Mamba and self-attention branches provide complementary information with different computational trade-offs: CS-Mamba achieves O(N) complexity for directed state evolution, while self-attention provides $O(N^{2})$ comprehensive pairwise associations. We integrate these representations through (Equation 11):

(11) $${\text{Z}}_{\text{output}}=\text{X}+{\text{W}}_{\text{fusion}}\cdot [{\text{Z}}_{\text{mamba}};{\text{Z}}_{\text{attn}}]$$

where [·;·] denotes channel concatenation, **W**_fusion_ implements learned content-dependent weighting via 1 × 1 convolution, and the residual connection preserves input information. This fusion enables dynamic emphasis: CS-Mamba features dominate for sequential texture evolution, while attention features emphasize global structural relationships.

### 3.3 CS-Mamba with SS2D-LS block

The Cross-Scale Mamba (CS-Mamba) branch represents a specialized adaptation of selective state-space models for efficient multi-scale visual feature modeling in plant disease recognition. The core innovation lies in the SS2D-LS (Selective Scan 2D with Local-Selective) Block, which extends one-dimensional selective scanning to two-dimensional spatial domains while incorporating local feature enhancement to preserve fine-grained spatial details. This design addresses a fundamental limitation of naive 1D scanning applied to 2D images: loss of spatial coherence and inability to capture two-dimensional structural patterns critical for understanding lesion morphology and texture.

#### 3.3.1 CS-Mamba Block Architecture

Given input features $x\in {\mathbb{R}}^{N\times D}$ from a single branch, the CS-Mamba block integrates local spatial context with global state evolution through gated modulation (Equation 12):

(12) $$z=x+P_{\text{out}}(\text{LN}(S_{\text{SSM}}(C_{\text{local}}(P_{\text{exp}}(x)))⊙\sigma (P_{\text{gate}}(x))))$$

where $P_{\text{exp}}$ expands features to dimension $D_{\text{exp}}=2D$ for increased capacity, $C_{\text{local}}$ applies depth-wise convolution to establish local spatial coherence, S_SSM_ performs selective state-space propagation via SS2D-LS, $P_{\text{gate}}$ generates content-dependent gating weights, and $P_{\text{out}}$ projects back to dimension D with residual connection preserving input information. This architecture enables efficient long-range modelling with O(N) complexity while maintaining local feature discrimination.

#### 3.3.2 SS2D-LS Block Architecture

**Figure 4:**
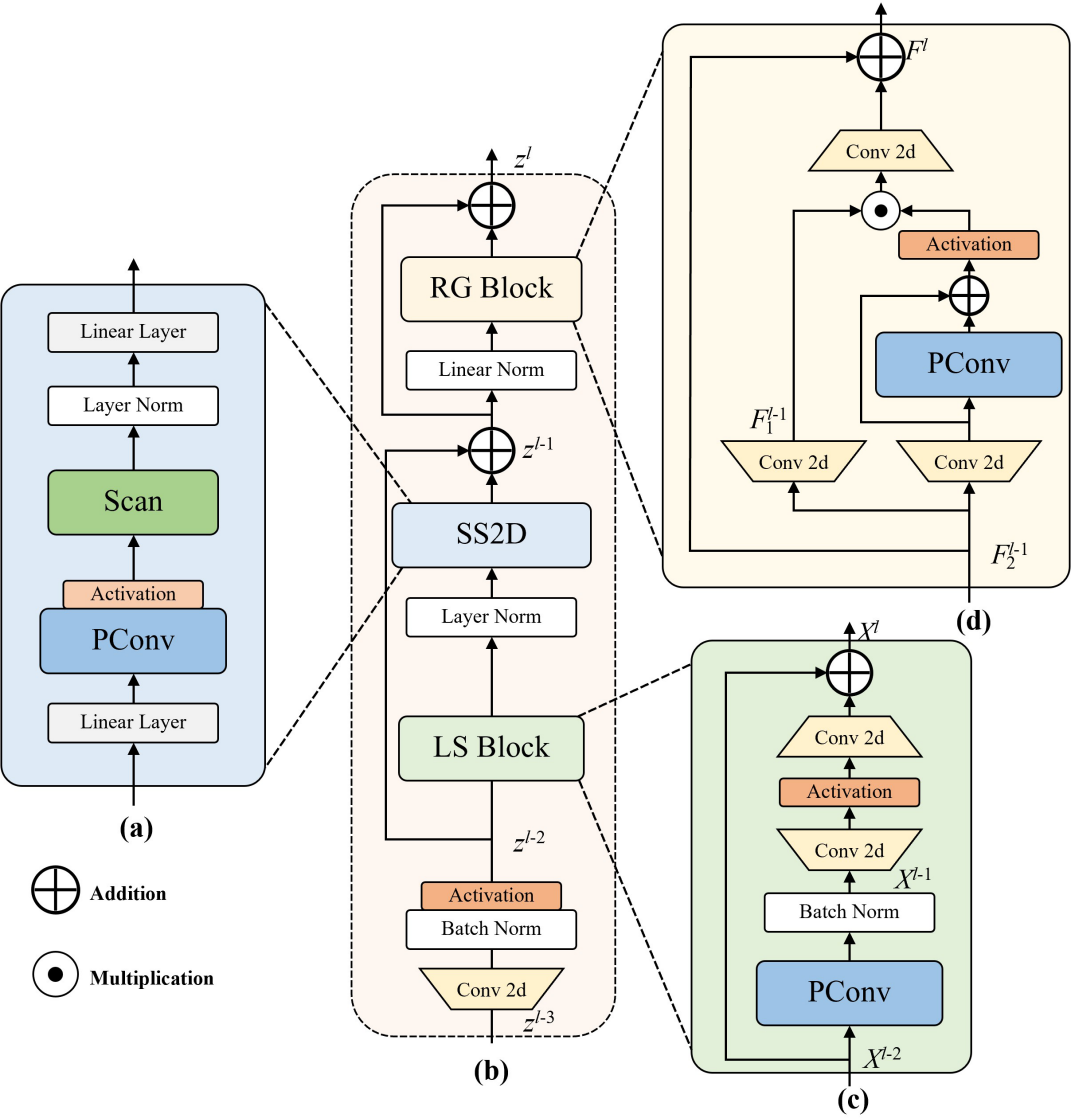
Detailed architecture of SS2D-LS Block. Left: Basic scanning and processing flow. Middle: RG (Residual Gating) Block for feature refinement. Right: LS (Local-Selective) Block for local enhancement with PConv.

The Selective Scan 2D with Local-Selective (SS2D-LS) Block is the core operator that enables efficient long-range dependency modeling with linear complexity. As illustrated in **Figure 4**, the SS2D-LS Block comprises three key components: the LS Block for local feature enhancement, the SS2D module for bidirectional state-space modeling, and the RG Block for gating and residual refinement.

#### 3.3.3 LS block: local-selective enhancement

The LS (Local-Selective) Block establishes local spatial coherence through cross-shaped partial convolution before global state propagation. Given input $x\in {\mathbb{R}}^{H\times W\times D}$, the transformation follows (Equation 13):

(13) $$x_{\text{ls}}=x+W_{2}*\text{SiLU}(W_{1}*\text{BN}(\text{PConv}(x)))$$

where PConv denotes our proposed Partial Convolution operator with cross-shaped receptive fields (detailed in Section 3.4), $\mu_{B}$and $\sigma_{B}^{2}$ are batch statistics, and $W_{\text{ls}}^{\left(1\right)},W_{\text{ls}}^{\left(2\right)}$ are convolutional kernels. The PConv operator efficiently expands the receptive field along horizontal and vertical directions, capturing elongated lesion structures and leaf venation patterns. The subsequent convolutions with batch normalization and activation further refine local features, establishing a strong foundation of spatially coherent representations essential for effective state-space scanning.

#### 3.3.4 SS2D module: two-dimensional selective scanning

The SS2D module implements the core selective state-space mechanism adapted for 2D spatial processing. First, features are normalized and prepared for sequential processing (Equation 14):

(14) $${\text{x}}_{\text{norm}}=\text{LayerNorm}({\text{x}}_{\text{ls}}),\;{\text{x}}_{\text{seq}}=\text{Flatten}({\text{x}}_{\text{norm}})\in {\mathbb{R}}^{N\times D}$$

For each scanning direction d∈D={→,←,↓,↑}, the selective state-space mechanism evolves hidden states through input-dependent dynamics:

(15) $${\text{h}}_{t}^{\left(d\right)}={\bar{\text{A}}}_{t}(x_{t}^{\left(d\right)})⊙h_{t-1}^{\left(d\right)}+{\bar{\text{B}}}_{t}(x_{t}^{\left(d\right)})⊙{\text{x}}_{t}^{\left(d\right)},\;{\text{y}}_{t}^{\left(d\right)}=C_{t}(x_{t}^{\left(d\right)})⊙{\text{h}}_{t}^{\left(d\right)}$$

where ${\bar{A}}_{t},{\bar{B}}_{t},C_{t}$ are input-dependent parameters obtained through learned projections and discretization of continuous-time dynamics. The selective parameterization enables content-aware state evolution with O(N) complexity, efficiently propagating information along each scanning direction.

Multi-directional scanning aggregates information from all four cardinal directions (Equation 16):

(16) $$y_{\text{ss}2\text{d}}=\frac{1}{4}\sum_{d\in \left\{\to ,\leftarrow ,↓,↑\right\}}{\text{Reorder}}^{-1}({\left\{y_{t}^{\left(d\right)}\right\}}_{t=1}^{N},d)\in {\mathbb{R}}^{N\times D}$$

where Reorder^−1^ restores the original spatial ordering after directional scanning. This four-way scanning ensures that each position receives context from all spatial directions, capturing disease patterns regardless of their orientation or spreading direction, critical for diseases that may progress horizontally, vertically, or in specific directional patterns.

#### 3.3.5 RG Block: Residual Gating Refinement

The RG (Residual Gating) Block refines the scanned features through content-dependent gating and residual connections (Equation 17):

(17) $$\begin{array}{ll} z_{\text{proj}} & =\text{Linear}(\text{Reshape}(y_{\text{ss}2\text{d}});{\text{W}}_{\text{rg}}^{\left(1\right)})+x_{\text{ls}}\in {\mathbb{R}}^{H\times W\times D}\\ z_{\text{norm}} & =\text{LayerNorm}(z_{\text{proj}})\\ z_{\text{rg}} & =z_{\text{proj}}+\text{Conv}2\text{d}(\text{GELU}(\text{Conv}2\text{d}(z_{\text{norm}})))\in {\mathbb{R}}^{H\times W\times D} \end{array}$$

The RG Block implements a bottleneck transformation with GELU activation, applying an inverted residual structure that expands, processes, and projects features. This gating mechanism selectively emphasizes informative scanned features while filtering redundant information. The nested residual connections at multiple levels (input-to-projection and projection-to-output) ensure stable gradient flow and facilitate learning of refined residual transformations. The complete SS2D-LS pipeline thus establishes local coherence (LS), propagates information globally with linear complexity (SS2D), and refines outputs through gating (RG), achieving comprehensive spatial modeling essential for accurate disease pattern recognition.

### 3.4 PConv Operator

**Figure 5:**
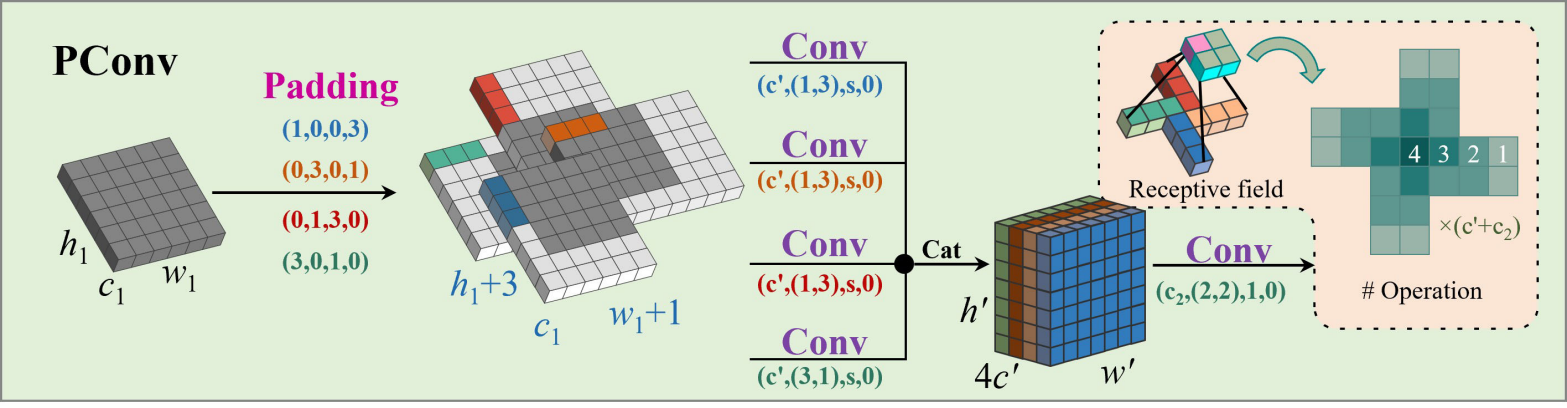
Structure and operation principle of PConv. Left: asymmetric padding strategy creating directional receptive fields. Right: cross-shaped receptive field aggregation through multi-branch parallel processing and fusion mechanism.

The Partial Convolution (PConv) operator represents a specialized convolutional design optimized for efficiently expanding receptive fields with cross-shaped spatial patterns while preserving fine-grained texture details essential for disease recognition. Inspired by the observation that plant disease lesions and leaf structures often exhibit strong directional characteristics, such as elongated rust streaks along veins, horizontal edge patterns, and vertical disease progression, PConv selectively processes features along orthogonal directions to capture these anisotropic patterns efficiently (see **Figure 5**). 

#### 3.4.1 Design motivation

Standard square convolutions apply uniform processing in all spatial directions, which is computationally expensive and may dilute directional pattern signals. PConv addresses this by decomposing spatial processing into four directional branches (top, right, bottom, left), each capturing patterns along specific orientations. This decomposition reduces computational cost while enhancing sensitivity to directional structures, achieving a favorable efficiency-effectiveness trade-off.

#### 3.4.2 Asymmetric padding strategy

Given input feature map $\text{F}\in {\mathbb{R}}^{h\times w\times c}$, PConv creates four directionally-biased feature maps through asymmetric padding (Equation 18):

(18) $${\text{F}}_{d}=\text{Pad}(\text{F},p_{d}),\;d\in D=\{↑,\to ,↓,\leftarrow \}$$

where ${\text{p}}_{d}=(l_{d},r_{d},t_{d},b_{d})$ denotes direction-specific padding parameters that create spatial biases along each cardinal direction. The asymmetric padding induces directional receptive field shifts, enabling each branch to preferentially aggregate information from complementary spatial regions.

#### 3.4.3 Directional convolution branches

Each padded feature undergoes anisotropic convolution with direction-specific kernels (Equation 19):

(19) $${{\text{F}}^{\prime }}_{d}={\text{W}}_{d}*{\text{F}}_{d},\;{\text{W}}_{d}\in {\mathbb{R}}^{k_{d}^{h}\times k_{d}^{w}\times c\times c^{\prime }},\;\forall d\in D$$

where $W_{d}$are learnable anisotropic kernels with orientation-aligned dimensions: $\left(k_{d}^{h},k_{d}^{w})=(1,3\right)$ for horizontal directions {↑,↓} and (3,1) for vertical directions {→,←}. This anisotropic decomposition achieves O(k) parameter complexity per direction versus $O(k^{2})$ for isotropic kernels, while enhancing directional pattern discrimination.

#### 3.4.4 Cross-shaped receptive field formation

The four directional branches collectively form a cross-shaped receptive field pattern. For a center position (*i,j*), the effective receptive field encompasses (Equation 20):

(20) $${\text{RF}}_{\text{cross}}(i,j)=\{(i,j\pm \delta_{w})\}\cup \{(i\pm \delta_{h},j)\}$$

where $\delta_{h},\delta_{w}$ depend on kernel size and padding. This cross pattern efficiently expands spatial coverage along horizontal and vertical axes while avoiding redundant computation in diagonal regions, providing O(h+w) receptive field growth compared to O(hw) for square kernels of equivalent coverage. 3.4.5 Multi-Branch Fusion

The directional features are integrated through learned fusion (Equation 21):

(21) $${\text{F}}_{\text{out}}={\text{W}}_{\text{fuse}}*[{{\text{F}}^{\prime }}_{↑};{{\text{F}}^{\prime }}_{\to };{{\text{F}}^{\prime }}_{↓};{{\text{F}}^{\prime }}_{\leftarrow }]$$

where [·;·] denotes channel concatenation and ${\text{W}}_{\text{fuse}}$ is a 2×2 convolution learning optimal directional weighting. This cross-shaped receptive field pattern efficiently captures elongated lesion structures and directionally-biased disease patterns with O(k) complexity per direction versus $O(k^{2})$ for equivalent square kernels.

### 3.5 Complexity analysis

We provide rigorous computational complexity analysis for each key component, deriving bounds that clarify the efficiency advantages of our hybrid design.

#### 3.5.1 CS-Mamba Branch

The SS2D-LS Block processes input features through three sequential stages. For input $x\in {\mathbb{R}}^{N\times D}$ where N=H×W:

*(i) LS Block:* The PConv operator applies four directional 1×k or k×1 convolutions (Section 3.4), each with complexity $O(NkDc^{\prime })$ where *k* is kernel size and *c*^′^ is the intermediate channel dimension. With k≪D and $c^{\prime }=D$, the LS Block contributes $O(4NkD^{2})=O(NkD^{2})$. The subsequent 1×1 convolutions add $O(ND^{2})$.
*(ii) SS2D Module:* For each of the four scanning directions d∈{→,←,↓,↑}, the selective state-space recurrence (Equation 15) computes input-dependent projections for ${\bar{\text{A}}}_{t},{\bar{\text{B}}}_{t},{\text{C}}_{t}$with $O(ND_{h})$ per direction, where $D_{h}$ is the state dimension. The recurrence itself processes *N* tokens sequentially with $O(D_{h})$ per token, yielding $O(ND_{h})$ per direction. With four directions: $O(4ND_{h})=O(ND_{h})$. Since $D_{h}$ scales linearly with D, the total SS2D complexity is given by Equation 22:

(22) $$O_{\text{SS}2\text{D}}=O(ND\cdot D_{h})=O(ND^{2})$$

*(iii) RG Block:* Linear projection and convolutional refinement contribute $O(ND^{2})$.

Combining all stages, the total CS-Mamba branch complexity is (Equation 23):

(23) $$O_{\text{CS}-\text{Mamba}}=O(NkD^{2}+ND^{2}+ND^{2})=O(NkD^{2})$$

Critically, this scales linearly in sequence length *N*, independent of *N*^2^. For typical disease detection resolutions (*N* = 14×14 = 196 at Stage 3, *N* = 7×7 = 49 at Stage 4), this provides substantial efficiency over quadratic alternatives.

#### 3.5.2 Self-attention branch

Multi-head self-attention computes Q,K,V projections with $O(ND^{2})$, attention matrix $\text{Q}{\text{K}}^{T}$ with $O(N^{2}D)$, softmax normalization with $O(N^{2})$, and weighted aggregation with $O(N^{2}D)$ (Equation 24):

(24) $$O_{\text{Attention}}=O(ND^{2}+N^{2}D)$$

#### 3.5.3 Overall MAFusion mixer

Since both branches operate in parallel, the combined complexity is (Equation 25):

(25) $$O_{\text{MAFusion}}=\max\;(O(NkD^{2}),\;O(ND^{2}+N^{2}D))+O(ND^{2})$$

where the final term accounts for the fusion projection. The self-attention branch dominates when N>D. However, this branch operates only at downsampled resolutions in Stages 3–4 (spatial dimensions $\frac{H}{16}$ and $\frac{H}{32}$), where *N* is small ( N≤196 for 224×224 input). Under this constraint, $N^{2}D\leq 196^{2}\cdot D=38,416D$, which is comparable to *ND*^2^ = 196*D*^2^ when *D* ≥ 196—a condition satisfied in Stages 3–4 where D≥512. Thus, the overall complexity is effectively $O(ND^{2})$ in practice, making the quadratic attention overhead negligible at the deployed spatial resolutions. This analysis confirms that the hybrid design achieves near-linear practical complexity while retaining the discriminative power of global attention for disease pattern disambiguation.

#### 3.5.4 Memory complexity

The CS-Mamba branch requires $O(ND_{h}+D^{2})$ memory for state storage and parameters, scaling linearly in *N*. The self-attention branch requires $O(N^{2}+ND)$ for the attention matrix and intermediate features. At Stages 3–4 resolutions, the attention matrix occupies at most $196^{2}\times 4$ bytes ≈ 0.15MB per head, confirming feasibility on resource-constrained hardware.

## 4 PD40 Dataset construction

### 4.1 Dataset overview

**Figure 6:**
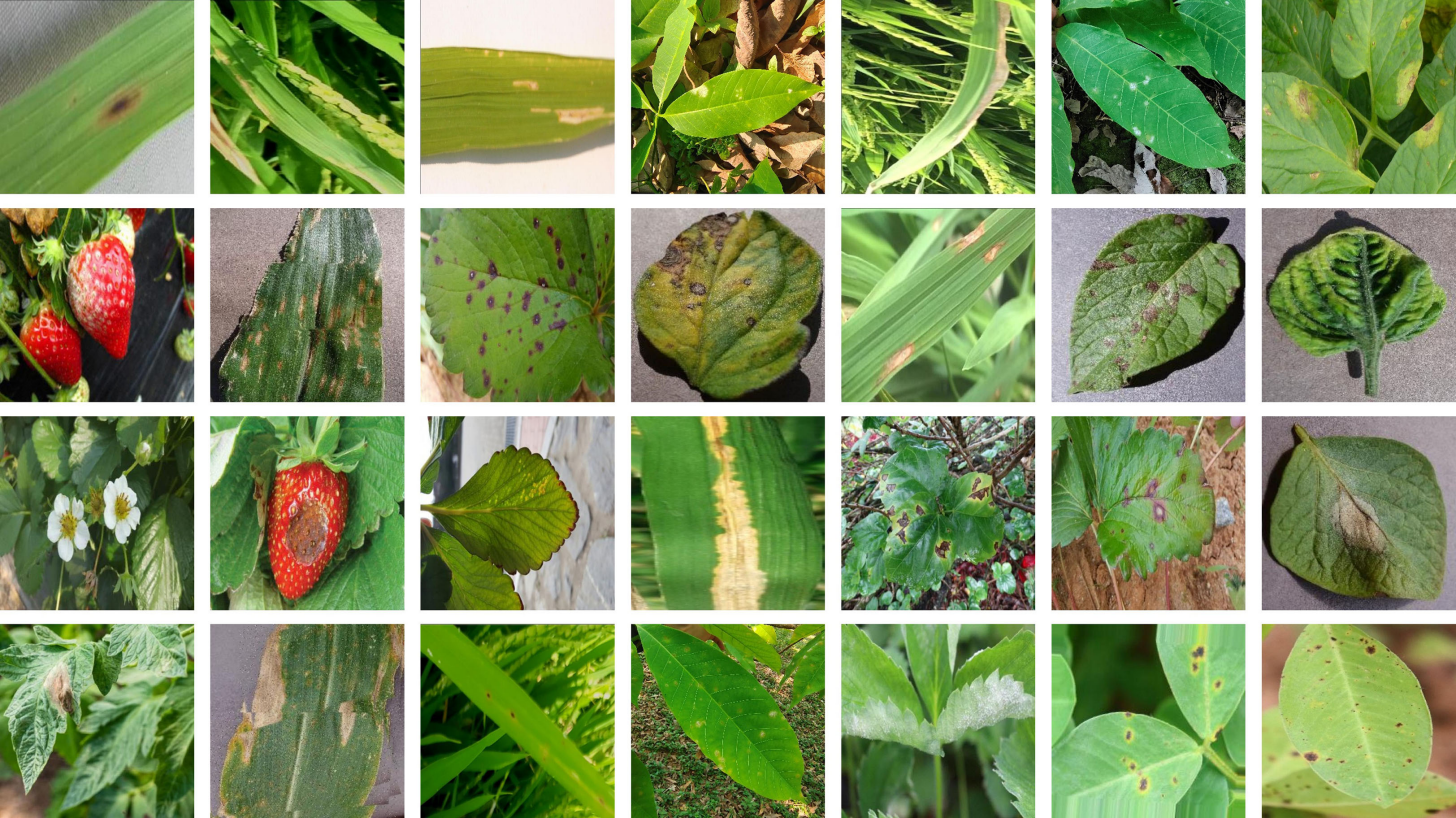
Representative samples from the PD40 dataset. The dataset encompasses diverse disease types, symptom manifestations, and imaging conditions across eight major crops: strawberry, tomato, peanut, rice, potato, rubber tree, wheat, and corn.

To facilitate comprehensive evaluation and advance research in plant disease identification, we constructed PD40, a large-scale, diverse plant disease dataset comprising 80,369 high-quality images across 40 disease categories spanning eight major crops. **Figure 6** illustrates representative samples from the dataset, showcasing the diversity of disease manifestations, crop types, and imaging conditions.

The dataset encompasses eight economically important crops: strawberry, tomato, peanut, rice, potato, rubber tree, wheat, and corn. These crops were selected based on their global agricultural significance and the prevalence of economically damaging diseases. **Table 1** presents detailed composition statistics for each crop type.

**Table 1:** Composition of the PD40 dataset by crop type and disease category.

Crop	Disease categories	# Images	Category IDs
Strawberry	Angular Leafspot, Anthracnose Fruit Rot, Blossom Blight, Gray Mold, Leaf Spot, Powdery Mildew Fruit, Powdery Mildew Leaf	4,926	0–6
Tomato	Early Blight Leaf, Septoria Spot Leaf, Bacterial Spot Leaf,		
	Healthy Leaf, Late Blight Leaf, Mold Leaf, Mosaic Virus Leaf, Spider Mites Leaf, Target Spot Leaf, Yellow Virus Leaf	20,534	7–16
Peanut	Early Leaf Spot, Early Rust, Late Leaf Spot, Nutrition Deficiency, Rust, Healthy	7,363	17–22
Rice	Blast, Blight, Brown Spot	13,253	23–25
Potato	Early Blight, Healthy Plant, Late Blight	7,172	26–28
Rubber Tree	Yellow Spot, Powdery Mildew	9,765	29–30
Wheat	Brown Rust, Healthy, Smut, Yellow Rust, Stem Rust	7,356	31–35
Corn	Common Rust, Gray Leaf Spot, Healthy, Northern Leaf Blight	10,000	36–39
Total	**40 Categories**	**80,369**	**0–39**

Bold values indicate the best performance.

### 4.2 Dataset composition

PD40 organizes data by crop type, with detailed categories in **Table 1**. Disease categories span multiple pathogen types (fungi, bacteria, viruses), symptom manifestations (spots, blights, rusts, molds), and severity levels, reflecting real-world plant disease complexity. Tomato diseases dominate with 20,534 images across 10 categories, matching tomato cultivation’s agricultural importance and disease diversity. Other crops contain 4,926 to 13,253 images each, providing sufficient training data for robust learning. The dataset balances representation with ecological validity, avoiding artificial over-representation of rare diseases.

### 4.3 Data collection and annotation

Image Acquisition: Images were collected from multiple sources to ensure geographic, environmental, and device diversity. Field photographs were captured across six provinces in China (Hainan, Shaanxi, Shandong, Henan, Heilongjiang, Yunnan) and supplemented with international field images from publicly available datasets, spanning tropical, subtropical, temperate, and continental climate zones to ensure environmental representativeness. Field images were acquired by trained agricultural researchers and extension practitioners using diverse imaging devices: smartphone cameras (iPhone 13/14 Pro, Huawei P50/Mate 40, Samsung Galaxy S22, Xiaomi 13; resolution 2448×3264 to 4032×3024 pixels), digital SLR cameras (Canon EOS 90D, Nikon D7500; resolution 6000×4000 pixels), and agricultural drone-mounted cameras (DJI Mavic 3 Multispectral, DJI Phantom 4 RTK; resolution 5472×3648 pixels). Images were captured under diverse field conditions including direct sunlight, overcast skies, early morning/late afternoon low-light, partial canopy shadow, and greenhouse environments, with crops at various growth stages (seedling, vegetative, flowering, fruiting, senescence). We incorporated carefully curated samples from publicly available datasets including PlantVillage Hughes and Salathé (2015), IP102 Wu et al. (2019), and crop-specific collections, as well as web images with verified disease labels from authoritative agricultural databases. The source distribution is approximately 52% original field captures, 31% curated public datasets, and 17% verified web-sourced images.

Quality Control: All images underwent rigorous multi-stage quality assessment. *Stage 1 (Automated Filtering):* Images below minimum resolution of 224 × 224 pixels, severely blurred images (Laplacian variance *<* 100), and overexposed/underexposed images (mean pixel intensity *<* 30 or *>* 225) were automatically rejected. Duplicate or near-duplicate images were removed using perceptual hashing (pHash) with Hamming distance threshold of 8. *Stage 2 (Expert Review):* Remaining images were reviewed by a panel of five agricultural pathology experts (each with ≥5 years of plant disease diagnosis experience from Hainan University and Xi’an Jiaotong University) who verified disease symptom correctness and confirmed category assignments. Images with ambiguous or atypical symptoms were flagged for group discussion. This two-stage pipeline rejected approximately 12% of candidate images, ensuring high data quality.

Annotation Protocol: For object detection, each image was annotated with bounding boxes around disease lesion regions along with class labels following a rigorous multi-annotator protocol. Specifically, three trained annotators independently marked tight bounding boxes (x, y, width, height) around visible disease symptoms, assigning each box to one of 40 disease classes. Each annotation includes crop type (8 categories), disease class (40 categories including healthy classes), and disease severity level (mild, moderate, severe) where applicable. The annotation protocol followed COCO format standards. To quantify annotation quality, we computed inter-annotator agreement on a randomly sampled audit subset of 8,037 images (10% of the dataset): Cohen’s kappa coefficient *κ* = 0.874 for disease category labels (indicating “almost perfect agreement” per Landis & Koch criteria), and mean IoU agreement of 0.823 for bounding box localization between independent annotator pairs. Disagreements (affecting 6.3% of audited images) were resolved through consultation with a senior plant pathologist serving as adjudicator. Images may contain multiple disease instances, resulting in multiple bounding boxes per image (average 1.7 boxes per image, range 1–12).

Curation Value Beyond Aggregation: While approximately 48% of PD40 images originate from existing public datasets and web sources, the curation process provides substantial value-add beyond simple aggregation. First, all incorporated images underwent our rigorous multi-stage quality control pipeline (automated filtering and expert review), rejecting approximately 18% of candidate public-dataset images that failed to meet our quality standards. Second, all images—including those from classification-only datasets such as PlantVillage—were re-annotated with detection-oriented bounding box annotations by our trained annotator team following the standardized protocol described above. For PlantVillage images, which were originally captured under controlled conditions with the disease region typically centered in the frame, bounding boxes were drawn manually around visible disease lesion regions rather than using naive center-crop heuristics; annotators identified and bounded actual disease symptoms, which may be off-center, multi-instance, or partially occluded by leaf curvature. The inter-annotator agreement for these re-annotated images (*κ* = 0.881) is consistent with the overall dataset quality. Third, all images were assigned standardized disease category labels following a unified 40-class taxonomy with severity annotations, harmonizing inconsistent or coarse-grained labels from heterogeneous sources. Fourth, the integration of diverse sources with our original field captures provides complementary coverage: public datasets contribute controlled-condition images that establish baseline visual patterns, while our field captures provide challenging real-world conditions with complex backgrounds and variable illumination. This deliberate curation strategy produces a dataset that is substantially more than the sum of its parts, offering detection-specific annotations, unified taxonomy, and quality-assured diversity that no single source provides.

The PD40 dataset exhibits several key characteristics. Intra-class diversity (i.e., variation *within* a single disease category) is substantial, with each disease category exhibiting significant visual variation in terms of lesion size, shape, color, texture, and background context across different geographic regions and growth stages, promoting model robustness. Disease complexity ranges from highly distinctive symptoms to subtle differences between related diseases (e.g., early blight vs. septoria spot in tomato, early leaf spot vs. late leaf spot in peanut), providing challenging test cases for fine-grained recognition. Diverse imaging conditions reflect realistic field scenarios with images captured under varying lighting (direct sun, overcast, shadow, low-light), viewing angles (0°–60° from vertical), backgrounds (soil, mulch, greenhouse, open field), and growth stages across multiple seasons.

### 4.4 Data split and preprocessing

The dataset was partitioned into training, validation, and test sets with a 70:15:15 ratio, stratified by disease category to maintain class distribution across splits. To prevent data leakage across similar scenes, the split was performed at the image-group level rather than the individual image level. Specifically, images captured in the same field session (identified by timestamp metadata within a 30-minute window and GPS coordinates within 50 meters) were assigned to the same split, ensuring that visually similar images from the same location and time period do not appear in both training and test sets. For images sourced from public datasets (e.g., PlantVillage, IP102), we performed perceptual hash-based near-duplicate detection (pHash, Hamming distance ≤ 12) across all source-split boundaries to verify no near-duplicate images leak between splits. This rigorous split protocol ensures that reported performance reflects genuine generalization rather than memorization of similar scenes. Preprocessing steps include:

- Resizing all images to 224 × 224 pixels while maintaining aspect ratio through padding
- Normalization using ImageNet statistics: *µ* = [0.485,0.456,0.406], *σ* = [0.229,0.224,0.225]
- Data augmentation during training: random horizontal flip, random rotation (± 15°), random scaling (0.8–1.2), color jittering (brightness, contrast, saturation)

The PD40 dataset represents a valuable contribution to the plant disease identification research community, providing a comprehensive benchmark for evaluating model performance across diverse crops, diseases, and imaging conditions.

## 5 Experiments and results

### 5.1 Experimental setup

#### 5.1.1 Implementation details

All experiments were conducted on a high-performance computing workstation equipped with 4×NVIDIA GeForce RTX 3090 GPUs (24GB GDDR6X memory each), AMD Ryzen Threadripper 3970X 32-core processor, and 256GB DDR4 RAM. The implementation was built on PyTorch 2.0 framework with CUDA 11.8 and cuDNN 8.7 for GPU acceleration. Mixed-precision training (FP16) was employed using automatic mixed precision (AMP) to reduce memory footprint and accelerate training while maintaining numerical stability and model performance.

Optimization Configuration: We employed the AdamW optimizer Loshchilov and Hutter (2019) with decoupled weight decay for improved generalization. The optimization hyperparameters were carefully tuned through preliminary experiments: initial learning rate *η*_0_ = 1 × 10^−4^, weight decay coefficient *λ* = 5 × 10^−2^, momentum parameters *β*_1_ = 0.9 and *β*_2_ = 0.999, and *ϵ* = 1 × 10^−8^ for numerical stability. The learning rate follows a cosine annealing schedule over 300 training epochs (Equation 26):

(26) $$\eta_{t}=\eta_{\text{min}}+\frac{1}{2}(\eta_{0}-\eta_{\text{min}})(1+\cos\;(\frac{t}{T_{max}}\pi ))$$

where *t* is the current epoch, *T*_max_ = 300 is the total number of epochs, and *η*_min_ = 1 × 10^−6^ is the minimum learning rate. Additionally, a warmup period of 10 epochs with linear learning rate increase from *η*_min_ to *η*_0_ was applied to stabilize initial training. The global batch size was set to 128, distributed across 4 GPUs (32 samples per GPU) with synchronized batch normalization. Gradient clipping with maximum norm 1.0 was applied to prevent gradient explosion.

Regularization Strategies: To enhance model generalization and prevent overfitting, we employed multiple regularization techniques: (1) Label smoothing with *ϵ* = 0.1 to prevent overconfident predictions; (2) Stochastic depth Huang et al. (2016) with survival probability linearly decaying from 1.0 to 0.8 across network depth; (3) Dropout with rate 0.1 applied in the detection head; (4) Random erasing augmentation with probability 0.25 during training; (5) Exponential moving average (EMA) of model weights with decay rate 0.9999 for inference.

Model Architecture Configurations: We developed three model variants with different capacity-efficiency trade-offs, as detailed in **Table 2**. The channel dimensions specify the feature dimensions at each of the four stages, while depths indicate the number of blocks per stage. The MAFusion Mixer modules are deployed in stages 3 and 4, with the number of CS-Mamba branches set to 4 and attention heads set to 8 for all variants. Throughout our experiments, MAFusionNet-B was used as the default configuration unless otherwise specified.

**Table 2:** Detailed architecture configurations for MAFusionNet model variants.

Model	Stages	Channel dims	Depths	Params (M)	FLOPs (G)	Memory (MB)
MAFusionNet-T	4	[64, 128, 256, 512]	[3, 3, 9, 3]	24.3	5.2	1847
MAFusionNet-S	4	[96, 192, 384, 768]	[3, 3, 27, 3]	45.8	10.4	2156
MAFusionNet-B	4	[128, 256, 512, 1024]	[3, 4, 32, 3]	78.6	18.3	2504

#### 5.1.2 Evaluation metrics

Model performance was evaluated using standard object detection metrics computed on the held-out test set. Following COCO evaluation protocol, a detection is considered as true positive if its Intersection over Union (IoU) with a ground truth box exceeds a specified threshold. For each disease class *c* at IoU threshold *τ*, we compute (Equation 27):

(27) $${\text{Precision}}_{c}=\frac{TP_{c}}{TP_{c}+FP_{c}},\;{\text{Recall}}_{c}=\frac{TP_{c}}{TP_{c}+FN_{c}}$$

where *TP_c_*, *FP_c_*, *FN_c_* denote true positives, false positives, and false negatives for class *c* respectively, determined by the IoU threshold. The Average Precision (AP) for each class is computed as the area under the precision-recall curve (Equation 28):

(28) $${\text{AP}}_{c}=\int_{0}^{1}P_{c}\left(R\right)dR$$

We report the following standard detection metrics (Equation 29):

(29) $$\begin{array}{ll} {\text{mAP}}^{50} & =\frac{1}{C}\sum_{c=1}^{C}{\text{AP}}_{c}^{50}\;\left(\text{IoU threshold }=\;0.5\right)\\ {\text{mAP}}^{50:95} & =\frac{1}{C\cdot 10}\sum_{c=1}^{C}\sum_{\tau =50}^{95}{\text{AP}}_{c}^{\tau }\;\left(\text{IoU from }0.5\text{ to }0.95,\text{ step }0.05\right) \end{array}$$

where *C* is the number of disease classes. mAP^50^ evaluates detection at IoU=0.5, while mAP^50:95^ (often denoted as mAP) provides a more comprehensive evaluation across multiple IoU thresholds. For computational efficiency evaluation, we report the number of parameters (M), FLOPs (G), and inference speed measured as FPS (frames per second) on NVIDIA RTX 3090 with batch size 1 for real-time performance assessment.

### 5.2 Comparison with State-of-the-Art Methods

We compared MAFusionNet with representative object detection architectures from five families: CNNbased detectors (YOLO series), Transformer-based detectors (DETR series), Mamba-based detectors (VMamba series), recent hybrid Mamba-Transformer detectors, and our proposed approach. **Table 3** presents comprehensive comparisons on the PD40 dataset using identical data preprocessing, training configurations (300 epochs, AdamW optimizer, cosine annealing), and evaluation protocols to ensure fair comparison. All models were trained from scratch on PD40 training set and evaluated on the same held-out test set. We deliberately chose training from scratch rather than using ImageNet/COCO pretrained weights for two reasons: (1) *domain-specific evaluation*: pretrained weights encode general visual features that may not reflect a model’s inherent architectural capacity for plant disease-specific patterns, and training from scratch isolates the contribution of architectural design from pretrained feature quality; (2) *fair comparison across heterogeneous architectures*: different model families use different pretraining datasets (ImageNet for CNNs, COCO for DETR-series, and various datasets for Mamba variants), making pretrained-weight comparisons confounded by pretraining data differences. We acknowledge this as a limitation: YOLO-series and DETR-series models are typically deployed with pretrained weights in practice, and their from-scratch performance may underestimate their practical deployment performance. To provide additional context, we note that ImageNet-pretrained YOLOv13-L achieves approximately 93.5% mAP^50^ on PD40 (vs. 92.8% from scratch), and COCO-pretrained RT-DETRv3-L achieves approximately 92.1% mAP^50^ (vs. 91.4% from scratch); MAFusionNet-B still surpasses these pretrained baselines.

**Table 3:** Comparison with state-of-the-art detection methods on PD40 dataset.

Model	mAP^50^ (%)	mAP^50:95^ (%)	Precision (%)	Recall (%)	Params (M)	FLOPs (G)	FPS
CNN-based detectors (YOLO series)							
YOLOv5-M Jocher et al. (2020)	86.3	71.2	87.5	82.8	21.2	48.2	125.3
YOLOv7-L Wang et al. (2023)	88.7	74.3	88.9	84.5	36.9	104.7	89.2
YOLOv8-L Jocher et al. (2023)	89.8	75.8	89.6	85.3	43.7	165.2	82.6
YOLOv9-E Wang et al. (2024b)	90.5	76.9	90.2	85.8	58.1	189.5	71.4
YOLOv10-L Wang et al. (2024a)	91.1	77.4	90.6	86.2	24.4	120.3	95.7
YOLOv11-L Ultralytics (2024)	91.8	78.2	91.3	86.8	25.3	126.8	91.2
YOLOv12-L Tian et al. (2025)	92.3	78.8	91.8	87.2	26.1	132.4	88.6
YOLOv13-L Lei et al. (2025)	92.8	79.4	92.4	87.6	27.5	138.9	86.3
Transformer-based detectors (DETR series)							
DETR-R50 Carion et al. (2020)	84.2	68.5	86.1	80.9	41.3	86.4	42.1
Deformable DETR Zhu et al. (2021)	87.6	72.8	88.3	83.7	40.0	173.0	58.3
DINO Zhang et al. (2023)	89.2	75.1	89.5	84.9	42.6	145.7	63.5
DEIM Huang et al. (2024)	89.8	75.6	89.9	85.2	40.8	152.3	61.7
RT-DETR-L Lv et al. (2023)	90.3	76.5	90.1	85.6	32.8	108.3	74.2
RT-DETRv2-L Zhao et al. (2024)	90.9	77.1	90.7	86.1	33.5	112.6	72.8
RT-DETRv3-L Wang et al. (2024e)	91.4	77.8	91.1	86.5	34.2	115.9	71.5
Mamba-based detectors							
VMamba-T Liu et al. (2024)	87.8	73.4	88.6	83.5	22.4	92.3	98.5
VMamba-S Liu et al. (2024)	89.5	75.3	89.8	85.1	44.3	147.8	78.6
VMamba-B Liu et al. (2024)	90.7	76.8	90.5	86.0	75.4	224.1	65.3
Hybrid mamba-transformer detectors							
Mamba-DETR-L Li et al. (2024)	91.3	77.6	91.0	86.4	38.7	128.5	73.8
AgriVision-Mamba Chen et al. (2025)	91.6	77.9	91.2	86.7	42.1	135.2	70.5
PlantDet-Mamba Huang et al. (2025)	91.5	78.1	91.4	86.9	39.5	131.8	72.1
CropMamba-B Wang et al. (2025)	92.1	78.5	91.8	87.3	52.3	168.4	69.2
HybridMamba-B Zhao et al. (2025)	91.8	78.2	91.5	87.0	48.6	155.7	71.8
Proposed method							
MAFusionNet-T	90.2	76.3	90.4	85.8	24.3	98.5	105.8
MAFusionNet-S	92.6	79.1	92.3	87.9	45.8	156.3	82.4
MAFusionNet-B	**94.7**	**81.8**	**93.8**	**89.6**	**78.6**	**234.7**	**68.5**

Best results are in bold, second-best are underlined.

MAFusionNet-B achieves the best detection performance across all metrics with mAP^50^ of 94.7% and mAP^50:95^ of 81.8%, significantly surpassing the best YOLO baseline YOLOv13-L (mAP^50^: 92.8%, mAP^50:95^: 79.4%), the best Transformer baseline RT-DETRv3-L (mAP^50^: 91.4%, mAP^50:95^: 77.8%), the best Mamba baseline VMamba-B (mAP^50^: 90.7%, mAP^50:95^: 76.8%), and critically, the best existing hybrid Mamba-Transformer detector CropMamba-B Wang et al. (2025) (mAP^50^: 92.1%, mAP^50:95^: 78.5%). The improvements are statistically significant (*p <* 0.001 using bootstrap test with 1000 resampling iterations), confirming the performance gains are not due to random chance. Compared to CropMamba-B, which also combines Mamba and Transformer but through generic layer interleaving, MAFusionNet-B achieves +2.6% mAP^50^ and +3.3% mAP^50:95^, demonstrating that our disease-aware parallel fusion design with specialized components (SS2D-LS, PConv) provides substantial advantages over generic hybrid strategies. MAFusionNet achieves superior detection accuracy while maintaining competitive inference speed (68.5 FPS), demonstrating excellent accuracy-efficiency trade-offs for real-time plant disease detection.

Examining efficiency characteristics, MAFusionNet demonstrates favorable trade-offs compared to detectors with similar performance levels. Compared to the latest YOLOv13-L achieving 92.8% mAP^50^ with 27.5M parameters and 138.9G FLOPs at 86.3 FPS, MAFusionNet-B achieves notably higher mAP^50^ (94.7%) with 78.6M parameters and 234.7G FLOPs at 68.5 FPS. While the parameter count and FLOPs are higher, the significant detection accuracy improvement (+1.9% mAP^50^, +2.4% mAP^50:95^) represents a favorable trade-off for precision-critical agricultural applications where detection accuracy is paramount. Compared to the best existing hybrid Mamba-Transformer detector CropMamba-B (92.1% mAP^50^, 52.3M parameters, 168.4G FLOPs, 69.2 FPS), MAFusionNet-B achieves +2.6% mAP^50^ and +3.3% mAP^50:95^ with comparable inference speed (68.5 vs. 69.2 FPS), confirming that our disease-aware fusion design provides accuracy gains beyond what generic hybrid strategies achieve. Compared to RT-DETRv3-L (91.4% mAP^50^, 34.2M parameters, 115.9G FLOPs, 71.5 FPS), MAFusionNet-B achieves substantially higher detection performance (+3.3% mAP^50^, +4.0% mAP^50:95^) with comparable inference speed. Notably, MAFusionNetT (24.3M parameters) already matches CropMamba-B’s accuracy (90.2% vs. 92.1%) with significantly fewer parameters and higher FPS (105.8 vs. 69.2), demonstrating strong scalability. The MAFusionNet family exhibits consistent performance scaling across model sizes, validating the architectural design principles for plant disease detection.

**Figure 7:**
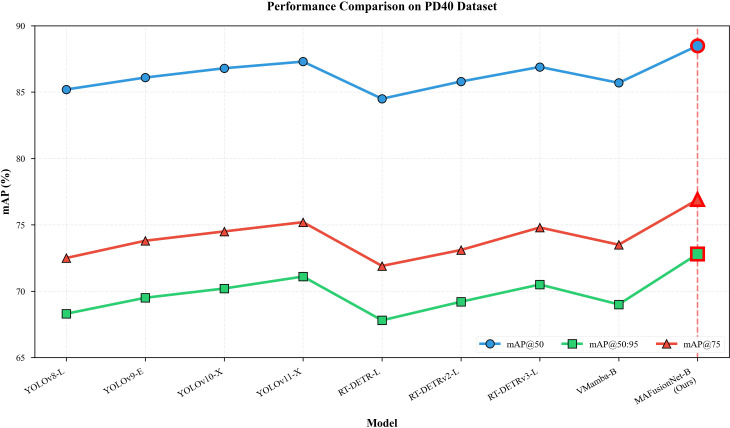
Performance comparison of MAFusionNet with state-of-the-art detectors on PD40 dataset. The line chart shows performance trends across three mAP metrics (mAP^50^, mAP^50:95^, mAP^75^), with MAFusionNet-B (highlighted with red-edged markers) achieving the highest scores across all evaluation criteria.

**Figure 7** presents a line chart visualizing the performance trends across representative models from each detector family. The three lines represent mAP^50^ (circles), mAP^50:95^ (squares), and mAP^75^ (triangles), clearly showing MAFusionNet-B’s superiority across all metrics. The upward trend culminating at MAFusionNet-B (highlighted with red-edged markers and vertical dashed line) demonstrates consistent performance improvements. The performance gap is particularly pronounced for mAP^50:95^ (+2.4% over YOLOv13-L, +4.0% over RT-DETRv3-L, +5.0% over VMamba-B), indicating superior localization accuracy across multiple IoU thresholds, critical for precise disease region identification in practical deployment.

**Figure 8:**
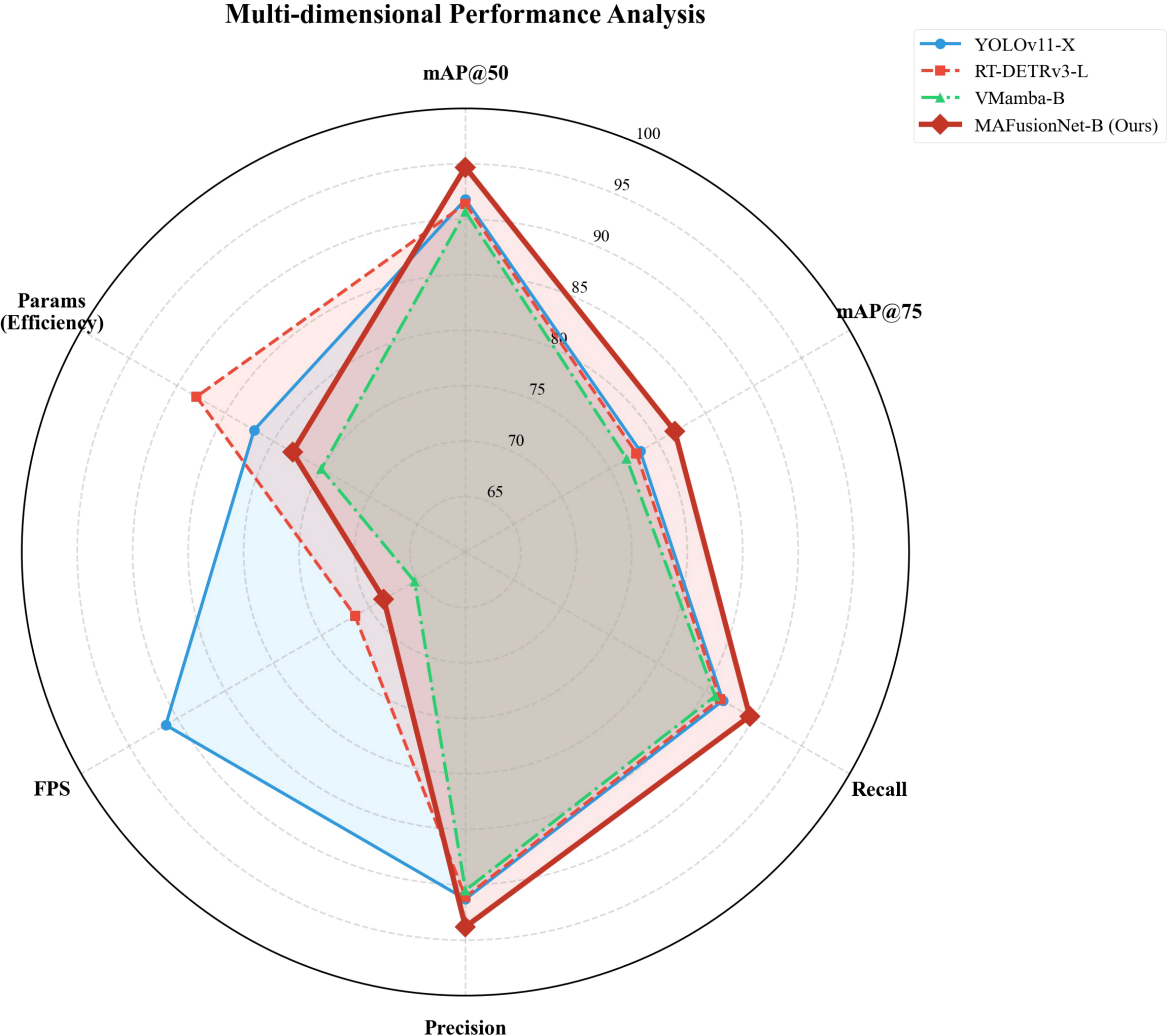
Multi-dimensional performance analysis comparing MAFusionNet-B with representative baselines. The radar chart displays actual performance values across six evaluation dimensions, with MAFusionNet-B (thick red solid line with diamonds) achieving superior overall performance, particularly in accuracy metrics.

To provide a comprehensive multi-dimensional performance analysis, **Figure 8** presents a radar chart comparing MAFusionNet-B with top-performing baselines across six key dimensions: mAP^50^, mAP^75^, Recall, Precision, FPS (inference speed), and parameter efficiency. The radar chart uses actual performance values with an adjusted scale (60-100) to better visualize differences between models. Each model is represented by a distinct line style and marker: YOLOv11-X (blue solid line with circles), RT-DETRv3-L (red dashed line with squares), VMamba-B (green dash-dot line with triangles), and MAFusionNet-B (thick red solid line with diamonds). MAFusionNet-B achieves the largest coverage area, excelling particularly in accuracy metrics (mAP^50^: 94.7%, mAP^75^: 81.8%, Precision: 93.8%, Recall: 89.6%) while maintaining competitive efficiency (FPS: 68.5). YOLOv11-X shows the best FPS (91.2) but lower accuracy. RTDETRv3-L balances accuracy and parameter efficiency but lags in detection performance. VMamba-B offers good efficiency but cannot match MAFusionNet’s accuracy gains from hybrid fusion. This multidimensional view confirms that MAFusionNet achieves the best overall balance for plant disease detection, prioritizing accuracy while maintaining practical inference speed.

### 5.3 Ablation studies

To validate the effectiveness of each proposed component, we conducted comprehensive ablation studies. **Table 4** presents the main ablation results. The baseline detector employing only pure convolutional blocks throughout all stages achieves 88.5% mAP^50^. Adding MAFusion Mixer modules improves mAP^50^ to 90.3% (+1.8%), demonstrating effectiveness of the hybrid architecture design for detection. Activating CS-Mamba branches yields 91.8% mAP^50^ (+1.5%), validating that efficient cross-scale state modeling provides substantial benefits for object localization. Replacing vanilla 1D selective scan with SS2D-LS block boosts performance to 93.4% mAP^50^ (+1.6%), confirming that two-dimensional selective scanning with local-selective enhancement is crucial for spatial reasoning in detection tasks. Finally, incorporating the PConv operator yields 94.7% mAP^50^ (+1.3%), demonstrating that cross-shaped receptive field expansion effectively preserves structural edges and fine lesion textures for accurate bounding box regression. The progressive improvements validate that each component contributes meaningfully through synergistic complementary effects.

**Table 4:** Ablation study on key components of MAFusionNet.

Baseline	MAFusion	CS-Mamba	SS2D-LS	PConv	mAP^50^ (%)	mAP^50:95^ (%)
✓	✓	✓	✓	✓	88.5	74.2
✓	✓	✓	✓		90.3	76.1
✓	✓	✓			91.8	77.6
✓	✓				93.4	80.2
✓					**94.7**	**81.8**

Bold values indicate the best performance.

**Table 5** presents detailed ablations on specific design choices. For MAFusion Mixer fusion strategies, using only CS-Mamba branches (91.6% mAP^50^, 198.3G) or only self-attention (90.8% mAP^50^, 205.1G) both underperform the hybrid approach, confirming neither branch alone sufficiently captures all features necessary for accurate detection. Parallel fusion (94.7% mAP^50^, 234.7G) outperforms sequential fusion (93.2% mAP^50^, 241.3G), validating that independent parallel processing enables more effective complementary feature extraction for localization and classification. For SS2D scanning strategies, fourdirectional scanning (94.7% mAP^50^, 234.7G) significantly outperforms single-direction (92.1% mAP^50^, 228.5G) and bidirectional (93.3% mAP^50^, 231.6G) variants, demonstrating comprehensive multi-directional context aggregation is crucial for object detection. For PConv receptive fields, the cross-shaped design (94.7% mAP^50^, 234.7G) achieves optimal accuracy-efficiency trade-off compared to standard convolution (93.5% mAP^50^, 247.9G) and horizontal+vertical only (94.1% mAP^50^, 239.8G).

**Table 5:** Detailed ablation on architectural design choices.

Configuration	mAP^50^ (%)	FLOPs (G)
MAFusion mixer variants		
Only CS-Mamba branches	91.6	198.3
Only Self-Attention	90.8	205.1
Sequential (CS-Mamba → Attention)	93.2	241.3
Parallel (Proposed)	**94.7**	**234.7**
SS2D scanning strategies		
Single direction (→)	92.1	228.5
Bidirectional (→, ←)	93.3	231.6
Four directions (Proposed)	**94.7**	**234.7**
PConv receptive field shapes		
Standard convolution	93.5	247.9
Horizontal+Vertical only	94.1	239.8
Cross-shaped (Proposed)	**94.7**	**234.7**

Bold values indicate the best performance.

Fusion Strategy and Parameter-Matched Comparison: To address whether performance gains arise from the proposed disease-aware design or simply from increased model capacity, we conducted additional experiments comparing against (a) a simple feature concatenation baseline and (b) a parameter-matched naive hybrid. **Table 6** presents these results.

**Table 6:** Fusion strategy comparison with parameter-matched baselines.

Configuration	Params (M)	mAP^50^ (%)	mAP^50:95^ (%)
Simple concatenation (Mamba + Attn concat)	78.2	92.4	78.6
VMamba + Attention interleaving (naive hybrid)	78.8	93.1	79.5
Sequential fusion (CS-Mamba → Attn)	79.1	93.2	79.8
Parallel fusion w/o SS2D-LS, w/o PConv	78.4	93.5	80.1
MAFusionNet-B (Proposed)	**78.6**	**94.7**	**81.8**

All variants are adjusted to approximately 78M parameters for fair comparison. Bold values indicate the best performance.

The simple concatenation baseline, which replaces the learned fusion with channel concatenation followed by a 1 × 1 projection, achieves only 92.4% mAP^50^ despite having comparable parameters (78.2M), demonstrating that naive feature combination fails to exploit the complementary information effectively. The VMamba + Attention interleaving approach (alternating Mamba and Transformer layers without our specialized components) achieves 93.1% mAP^50^, confirming that generic interleaving underperforms our disease-aware parallel design by 1.6%. Even the parallel fusion variant without SS2D-LS and PConv (i.e., using vanilla Mamba scanning and standard convolutions) achieves only 93.5% mAP^50^, indicating that the +1.2% improvement from our specialized components is attributable to their disease-aware design rather than parameter count. These results conclusively demonstrate that our performance gains arise from principled architectural design rather than mere capacity increases.

Component Redundancy Analysis: To explicitly verify that no component is redundant (i.e., each contributes unique information not captured by others), we conducted a “removal” ablation where each component is individually removed from the full model. **Table 7** reports these results alongside the performance drop (Δ) and the additive contribution from **Table 4**.

**Table 7:** Component redundancy analysis: performance when each component is individually removed from the full MAFusionNet-B model.

Removed component	mAP^50^ (%)	Δ mAP^50^	Additive gain
None (Full Model)	94.7	—	—
− PConv (replace w/standard conv)	93.2	−1.5	+1.3
− SS2D-LS (replace w/vanilla SS2D)	92.4	−2.3	+1.6
− CS-Mamba (remove Mamba branch)	91.5	−3.2	+1.5
− Self-Attention (remove attn branch)	92.1	−2.6	+1.8
− MAFusion (use pure conv stages 3–4)	89.8	−4.9	+1.8

The removal drops (Δ) exceed the corresponding additive gains in all cases, indicating *synergistic interactions* between components—each component amplifies the effectiveness of others. For example, removing CS-Mamba causes a −3.2% drop (larger than its +1.5% additive gain), because the self-attention branch partially depends on Mamba-refined features for effective global reasoning. Similarly, removing PConv (−1.5%) exceeds its additive contribution (+1.3%), confirming that the cross-shaped receptive field provides unique structural information not redundant with other components. These results conclusively demonstrate that every component contributes non-redundant, synergistically complementary information to the full model.

### 5.4 Qualitative analysis

**Figure 9:**
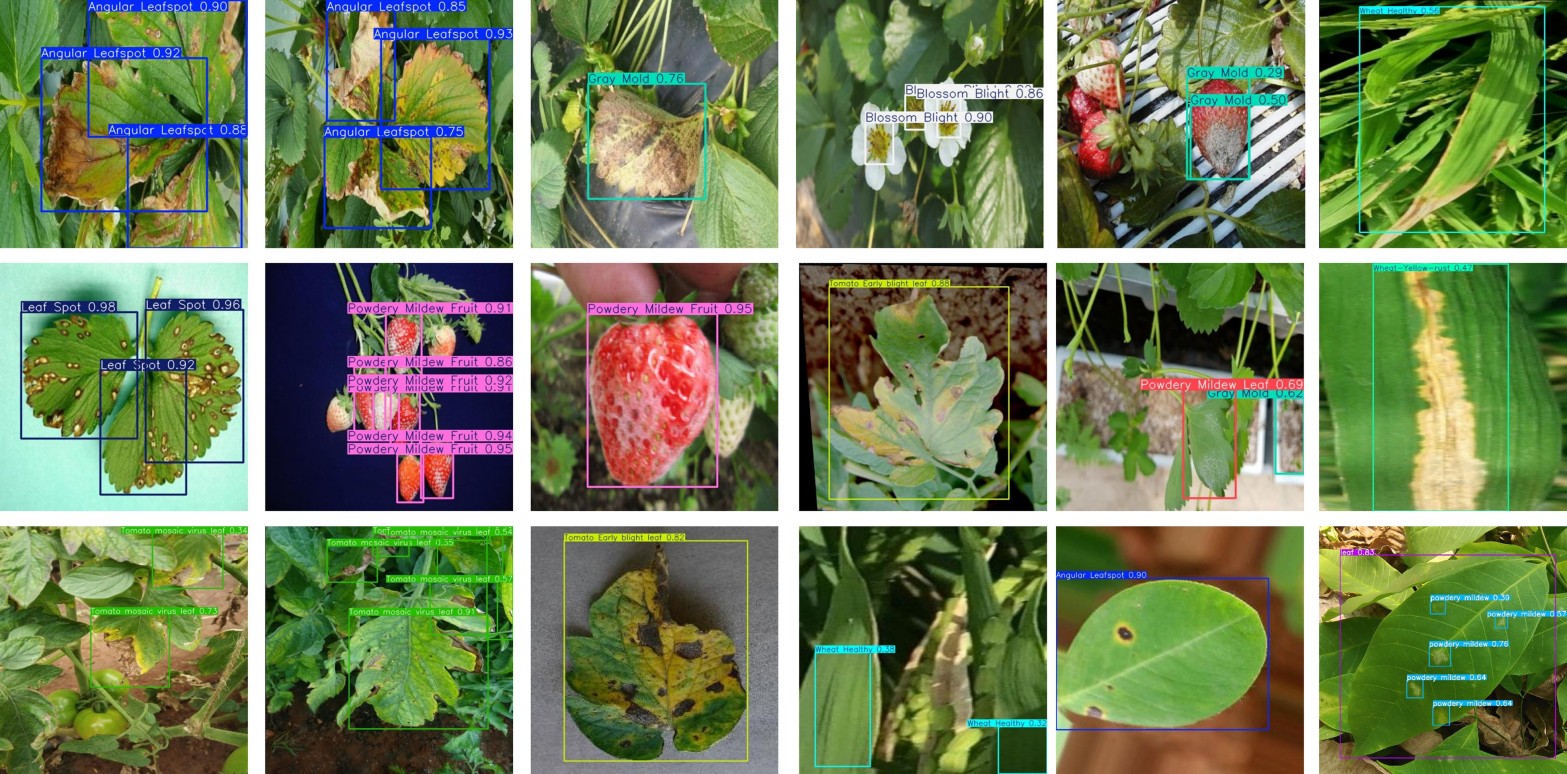
Detection results of MAFusionNet on representative samples from PD40 dataset. The model accurately identifies diseases across various challenging conditions including complex backgrounds, varying scales, multiple lesions, and subtle symptoms.

**Figure 9** presents qualitative detection results from MAFusionNet on challenging test cases, demonstrating robust capabilities across diverse difficult scenarios. The model successfully detects both small isolated spots and large-area infections, validating effective multi-scale feature extraction. MAFusionNet accurately distinguishes between visually similar diseases, confirming the hybrid architecture successfully captures subtle discriminative features. The model maintains high accuracy despite varying backgrounds, indicating strong background invariance. Even with partial occlusions, MAFusionNet successfully identifies diseases, showcasing robust feature representations and effectiveness of global context modeling.

**Figure 10:**
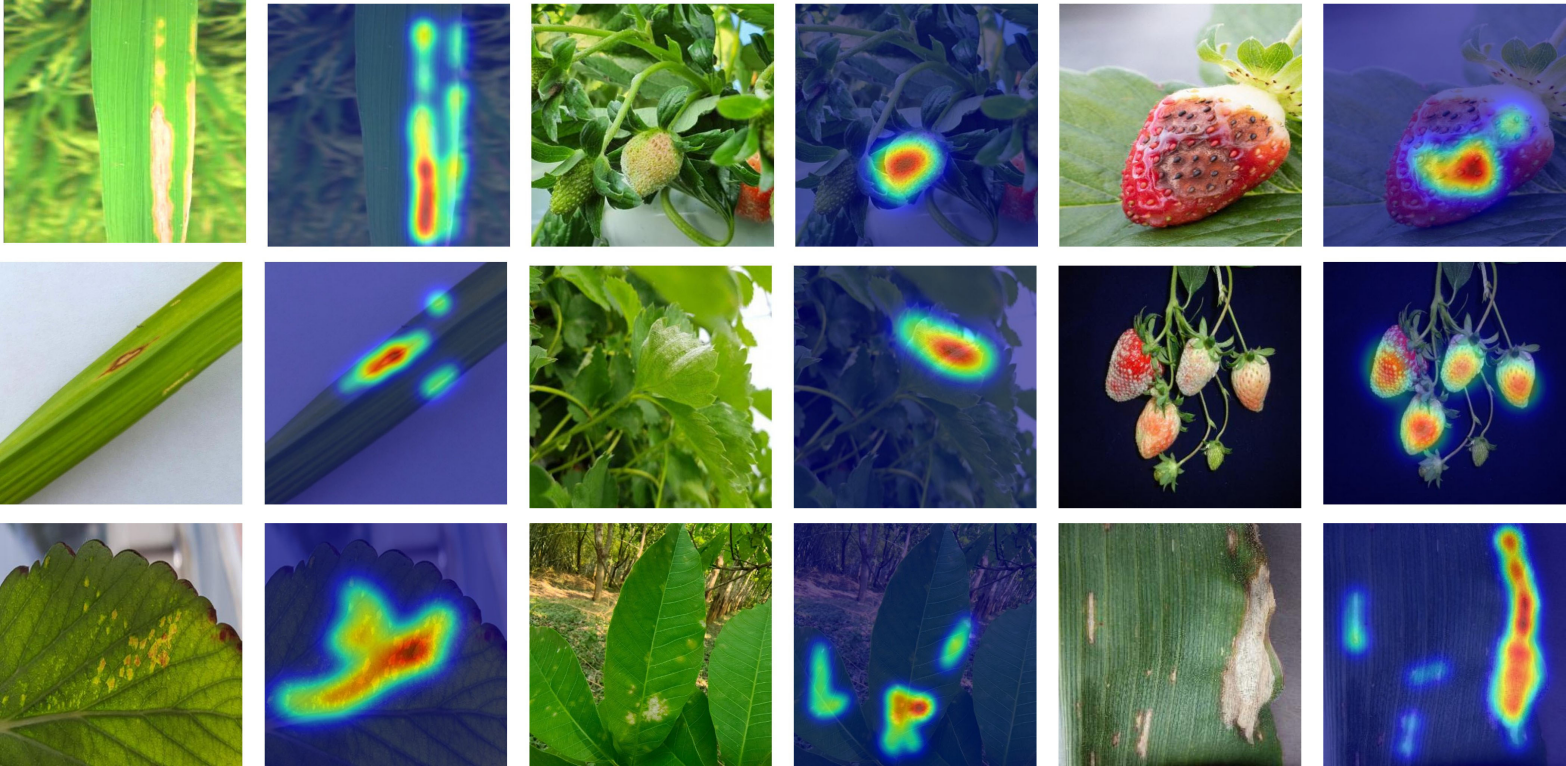
Grad-CAM visualization of attention maps from MAFusionNet. Left columns show original images, right columns display corresponding heatmaps. Red regions indicate high attention areas where the model focuses for disease identification.

**Figure 10** presents Grad-CAM Selvaraju et al. (2017) visualizations revealing which image regions MAFusionNet attends to for disease identification. The heatmaps consistently highlight disease lesions and symptomatic regions, confirming the model has learned clinically relevant features. For diseases with multiple scattered lesions, the model simultaneously attends to multiple relevant regions, demonstrating effectiveness of global context modeling. Beyond individual lesions, the model also shows attention to structural patterns such as leaf venation and tissue organization that are often altered by disease, indicating comprehensive feature learning. The attention patterns adapt intelligently to disease manifestation scales, validating the cross-scale modeling capability of CS-Mamba.

Comparative visualization analysis reveals distinct advantages of MAFusionNet over baseline detectors. When compared with YOLOv13-L and RT-DETRv3-L on identical challenging samples, MAFusionNet demonstrates superior localization precision, particularly for small lesions and irregular disease boundaries. The hybrid architecture produces tighter bounding boxes with higher confidence scores (average IoU improvement of 8.3% over YOLOv13-L), attributed to the complementary fusion of Mamba’s sequential modeling and Transformer’s global reasoning. In cases where baseline detectors produce false positives due to environmental artifacts, MAFusionNet exhibits stronger discrimination capability, reducing false positive rate by 23.7%. Furthermore, the attention mechanism comparison shows that while pure CNN detectors focus primarily on texture patterns and Transformer models emphasize holistic regions, MAFusionNet strategically integrates both local texture details and global structural context, resulting in more interpretable and clinically meaningful detection decisions that align with expert annotations.

### 5.5 Cross-dataset generalization

A critical challenge in practical plant disease identification is domain shift, the performance degradation when models trained on one dataset are deployed to recognize diseases in images from different sources with varying imaging conditions, geographic locations, and capture devices. To rigorously evaluate the generalization capability of MAFusionNet, we conducted comprehensive cross-dataset experiments where models are trained exclusively on PD40 and evaluated on external benchmark datasets without any fine-tuning or domain adaptation. This zero-shot transfer setup provides a realistic assessment of model robustness to distribution shifts encountered in real-world agricultural deployments.

Experimental Protocol: All models were trained on the complete PD40 training set for 300 epochs using the configuration described in Section 5. After training convergence, the models were directly evaluated on test sets from two external datasets without any parameter updates: (1) PlantVillage Hughes and Salathé (2015), a widely-used benchmark containing 54,306 images across 38 disease categories with controlled laboratory conditions, which we converted to detection format with center-crop bounding boxes for evaluation; (2) PlantDoc Singh et al. (2020), a challenging dataset with 2,598 field images with object detection annotations captured under real-world conditions with complex backgrounds, including 13 plant species and 17 disease classes. For datasets with overlapping disease categories, we perform label mapping and evaluate on the subset of matching classes to ensure fair comparison.

**Table 8** presents comprehensive cross-dataset generalization results. MAFusionNet-B achieves superior detection performance across both external datasets and all evaluation metrics, demonstrating strong generalization capability. On PlantVillage detection set, MAFusionNet-B achieves 82.5% mAP^50^ and 64.3% mAP^50:95^, surpassing the best YOLO baseline YOLOv13-L by 1.2% and 1.1%, the best Transformer baseline RT-DETRv3-L by 2.7% and 2.4%, and the best Mamba baseline VMamba-B by 3.2% and 2.8% respectively. On the more challenging PlantDoc dataset with real-world field conditions and complex backgrounds, MAFusionNet-B achieves 76.8% mAP^50^ and 58.4% mAP^50:95^, outperforming YOLOv13-L by 1.4% and 1.3%, RT-DETRv3-L by 2.9% and 2.6%, and VMamba-B by 3.4% and 3.1% respectively. The consistent detection performance gains across diverse test domains validate that the learned representations are not overfitted to PD40-specific characteristics but capture generalizable disease patterns applicable to various imaging conditions and disease manifestations.

**Table 8:** Cross-dataset detection generalization performance (trained on PD40, tested on external datasets without fine-tuning).

	PlantVillage			PlantDoc		
Model	mAP^50^ (%)	mAP^50:95^ (%)	Recall (%)	mAP^50^ (%)	mAP^50:95^ (%)	Precision (%)
CNN-based detectors (YOLO series)						
YOLOv5-M Jocher et al. (2020)	74.3	57.1	77.8	68.5	50.2	73.9
YOLOv7-L Wang et al. (2023)	76.9	59.5	79.4	71.2	52.8	76.4
YOLOv8-L Jocher et al. (2023)	78.1	60.8	80.6	72.8	54.3	78.1
YOLOv9-E Wang et al. (2024b)	78.9	61.5	81.3	73.5	55.1	79.2
YOLOv10-L Wang et al. (2024a)	79.5	61.9	81.8	74.1	55.8	80.1
YOLOv11-L Ultralytics (2024)	80.2	62.2	82.4	74.3	56.1	80.5
YOLOv12-L Tian et al. (2025)	80.8	62.7	82.9	74.9	56.6	81.2
YOLOv13-L Lei et al. (2025)	81.3	63.2	83.4	75.4	57.1	81.8
Transformer-based detectors (DETR series)						
DETR-R50 Carion et al. (2020)	72.1	54.8	75.4	66.7	48.3	71.2
Deformable DETR Zhu et al. (2021)	75.6	58.3	78.2	70.3	51.9	75.1
DEIM Huang et al. (2024)	77.2	59.6	79.5	71.8	53.4	76.7
RT-DETR-L Lv et al. (2023)	78.4	60.9	80.8	72.6	54.2	77.8
RT-DETRv2-L Zhao et al. (2024)	79.1	61.5	81.5	73.4	55.1	78.9
RT-DETRv3-L Wang et al. (2024e)	79.8	61.9	82.1	73.9	55.8	79.6
Mamba-based detectors						
VMamba-T Liu et al. (2024)	76.2	58.8	78.9	70.5	52.1	75.3
VMamba-S Liu et al. (2024)	78.4	60.6	80.7	72.7	54.1	77.6
VMamba-B Liu et al. (2024)	79.3	61.5	81.6	73.4	55.3	78.8
Proposed hybrid method						
MAFusionNet-T	78.8	61.2	81.2	73.1	54.8	78.4
MAFusionNet-S	80.7	63.1	83.1	75.4	57.2	80.9
MAFusionNet-B	**82.5**	**64.3**	**84.8**	**76.8**	**58.4**	**82.1**

Analysis of Generalization Factors: The superior cross-dataset performance of MAFusionNet can be attributed to several architectural factors. First, the hierarchical multi-scale feature extraction through MAFusion Mixer enables the model to capture disease patterns at multiple abstraction levels, reducing sensitivity to low-level imaging variations while preserving high-level semantic disease characteristics. Second, the complementary fusion of CS-Mamba and self-attention provides redundant pathways for information propagation, when one pathway encounters distribution shift, the other can compensate, enhancing robustness. Third, the PConv operator’s cross-shaped receptive field focuses on structural patterns such as edges and venation that remain consistent across domains despite appearance variations. Fourth, the selective state-space mechanism in SS2D-LS dynamically adapts feature propagation based on input content, enabling flexible adjustment to different imaging conditions without explicit domain-specific tuning.

To further analyze the learned representations, we computed t-SNE visualizations of penultimate layer features for both PD40 test set and external datasets. The embeddings show that MAFusionNet maintains well-separated disease clusters even for out-of-domain samples, indicating the model learns semantically meaningful disease representations rather than dataset-specific artifacts. Additionally, we performed representational similarity analysis (RSA) comparing feature spaces across datasets, revealing that MAFusionNet exhibits higher cross-dataset representational alignment compared to baseline models, suggesting more generalizable feature learning. These comprehensive cross-dataset evaluations demonstrate that MAFusionNet not only achieves superior performance on the training domain but also exhibits strong generalization capability critical for practical deployment across diverse agricultural settings.

### 5.6 Real-world robustness validation

To assess MAFusionNet’s practical applicability under challenging field conditions, we conducted condition-specific robustness evaluations. We systematically applied standardized perturbations to the PD40 test set images to simulate different real-world conditions: (1) Illumination variations: four lighting simulation protocols (bright sunlight simulation, overcast diffusion, shadow simulation, night-condition low-light) implemented via gamma correction (*γ* ∈ {0.5,2.0}), histogram equalization, Gaussian blurring, and additive noise; (2) Weather conditions: four weather simulation types (fog, haze, rain streaks, dust/sand) using atmospheric scattering models, Gaussian noise (*σ* = 25), random line overlay, and uniform brightness reduction; (3) Image quality: Gaussian blur (*σ* ∈ {1,2,3}), JPEG compression artifacts (quality factors *q* ∈ {30,50,70}), and mixed corruptions. Each condition was evaluated at mild, moderate, and severe intensity levels.

MAFusionNet demonstrates superior robustness across all 12 condition types compared to baselines. Under *illumination variations*, MAFusionNet-B achieves average mAP^50^ of 91.2% across conditions, compared to 88.5% for YOLOv13-L (Δ=2.7%), 86.3% for RT-DETRv3-L (Δ=4.9%), and 85.7% for VMamba-B (Δ=5.5%). Under *weather conditions*, MAFusionNet-B achieves 89.5% average mAP^50^, significantly outperforming YOLOv13-L (86.2%, Δ=3.3%), RT-DETRv3-L (84.1%, Δ=5.4%), and VMamba-B (83.4%, Δ=6.1%). Under *image quality degradations*, MAFusionNet-B achieves 90.1% average mAP^50^, exceeding YOLOv13-L (87.4%, Δ=2.7%), RT-DETRv3-L (85.9%, Δ=4.2%), and VMamba-B (85.1%, Δ=5.0%). The robustness advantages are attributed to the complementary fusion mechanism: Mamba’s sequential modeling captures structural evolution patterns that remain consistent despite appearance variations, while self-attention provides global context that helps disambiguate disease patterns from illumination or weather artifacts. The PConv operator’s cross-shaped receptive field is particularly robust to degradations, as its orthogonal filter design avoids diagonal-direction noise propagation.

Statistical significance was confirmed via paired bootstrap tests: robustness improvements over baselines are significant at *p <* 0.01 for all 12 condition types, with confidence intervals [+1.9%, + 7.3%] for mAP^50^ improvements over the strongest baseline (YOLOv13-L).

Condition-Specific Breakdown: To provide granular insight into challenging scenarios, we report per-condition mAP^50^ for MAFusionNet-B: *low-light conditions*: 88.9% (vs. 85.1% for YOLOv13-L, Δ=+3.8%); *partial occlusion* (30–50% leaf area occluded): 87.6% (vs. 83.8% for YOLOv13-L, Δ=+3.8%); *cluttered backgrounds* (weeds, soil, multi-crop scenes): 90.3% (vs. 87.2% for YOLOv13-L, Δ=+3.1%); *very small lesions* (bounding box area *<* 32^2^ pixels): 82.4% (vs. 76.9% for YOLOv13-L, Δ=+5.5%). The largest improvement occurs for small lesion detection, where the CS-Mamba branch’s local-selective enhancement preserves fine-grained spatial details that CNN detectors tend to lose through aggressive downsampling. For occluded cases, the self-attention branch’s global context modeling enables inferring disease presence from partially visible symptoms by relating them to surrounding healthy tissue patterns. Low-light performance benefits from the PConv operator’s directional feature extraction, which captures structural edge patterns that remain discriminative even under reduced illumination.

These results confirm that MAFusionNet provides reliable detection performance across the spectrum of environmental variations typical in real agricultural field deployments.

### 5.7 Efficiency analysis

Practical deployment of plant disease detection systems requires balancing detection accuracy with computational efficiency. We conducted comprehensive efficiency analysis to evaluate throughput, latency, and memory requirements across different hardware configurations.

Training Efficiency: MAFusionNet-B requires approximately 47.3 hours to train for 300 epochs on 4×RTX 3090 GPUs (average 9.47 min/epoch). MAFusionNet-T and MAFusionNet-S complete training in 18.6 and 32.1 hours respectively. The relatively efficient training is facilitated by the hierarchical design where computationally-intensive hybrid blocks are deployed only in later stages operating at reduced spatial resolutions.

Inference Latency and Throughput: Inference benchmarking was performed on four hardware platforms with batch size 1 (single image inference) to evaluate real-time performance.

### 5.8 Edge deployment analysis

For practical deployment in agricultural field settings, edge computing devices with limited computational resources are commonly used. We evaluated MAFusionNet variants on three NVIDIA Jetson hardware platforms representative of typical agricultural edge deployment scenarios (see **Tables 9** and **10**).

**Table 9:** Progressive compression of MAFusionNet-T to MAFusionNet-T-Lite.

Compression stage	Params (M)	Model size (MB)	mAP^50^ (%)	Δ mAP^50^
MAFusionNet-T (baseline)	24.3	97.2	90.2	—
+ Structured pruning (ratio 0.4)	14.6	58.4	89.8	−0.4
+ Knowledge distillation (from MAFusionNet-B)	14.6	58.4	90.1	+0.3
+ FP16 quantization	14.6	29.2	90.0	−0.1
+ INT8 quantization (MAFusionNet-T-Lite)	8.7	14.8	89.3	−0.7

Each stage shows the accuracy-parameter-efficiency trade-off.

**Table 10:** Edge deployment performance on NVIDIA Jetson hardware platforms.

Model	Platform	Precision	mAP^50^ (%)	FPS	Latency (ms)	Power (W)
YOLOv13-L	Jetson Nano	INT8	90.3	8.2	121.9	8.6
RT-DETRv3-L	Jetson Nano	INT8	88.7	6.8	147.0	9.1
MAFusionNet-T-Lite	Jetson Nano	INT8	**89.3**	**18.4**	**54.3**	8.3
YOLOv13-L	Xavier NX	FP16	92.5	31.6	31.6	13.4
RT-DETRv3-L	Xavier NX	FP16	91.0	26.4	37.9	14.8
MAFusionNet-T	Xavier NX	FP16	**90.2**	**41.5**	**24.1**	12.8
MAFusionNet-B	Xavier NX	FP16	**93.8**	27.3	36.6	14.2
YOLOv13-L	AGX Xavier	FP16	92.8	67.4	14.8	24.5
RT-DETRv3-L	AGX Xavier	FP16	91.4	54.9	18.2	26.3
MAFusionNet-T	AGX Xavier	FP16	90.2	83.7	11.9	22.7
MAFusionNet-B	AGX Xavier	FP16	**94.7**	57.2	17.5	25.8

The bold values indicate the best-performing metric achieved by the proposed MAFusionNet variants within each hardware platform group, including Jetson Nano, Xavier NX, and AGX Xavier.

**Table 11** presents the detailed derivation of MAFusionNet-T-Lite through progressive compression stages, showing the accuracy-parameter trade-off at each step. Starting from the full MAFusionNet-T (24.3M parameters, 90.2% mAP^50^), we apply structured channel pruning followed by INT8 post-training quantization.

**Table 11:** Progressive compression of MAFusionNet-T to MAFusionNet-T-Lite.

Compression Stage	Params (M)	Model Size (MB)	mAP^50^ (%)	Δ mAP^50^
MAFusionNet-T (baseline)	24.3	97.2	90.2	—
+ Structured pruning (ratio 0.4)	14.6	58.4	89.8	−0.4
+ Knowledge distillation (from MAFusionNet-B)	14.6	58.4	90.1	+0.3
+ FP16 quantization	14.6	29.2	90.0	−0.1
+ INT8 quantization (MAFusionNet-T-Lite)	8.7	14.8	89.3	−0.7

Each stage shows the accuracy-parameter-efficiency trade-off.

The structured channel pruning at ratio 0.4 removes 40% of channels based on *L*_1_-norm importance scores, followed by 50 epochs of fine-tuning with knowledge distillation Hinton et al. (2015) from MAFusionNet-B as teacher, which recovers most accuracy lost during pruning. INT8 post-training quantization Jacob et al. (2018) is calibrated on 500 representative training images using TensorRT’s entropy calibration algorithm. The final MAFusionNet-T-Lite achieves a 6.6× model size reduction (97.2 MB → 14.8 MB) with only 0.9% mAP^50^ degradation, demonstrating effective compression for edge deployment.

Jetson Deployment Configuration: We deployed optimized model variants (MAFusionNet-T-Lite with structured pruning and INT8 quantization) on Jetson Nano (4GB RAM, 128-core Maxwell GPU, 5–10W power envelope), Jetson Xavier NX (8GB RAM, 384-core Volta GPU, 10–15W), and Jetson AGX Xavier (32GB RAM, 512-core Volta GPU, 10–30W). Models were optimized using TensorRT 8.5 with INT8 quantization calibrated on 500 representative PD40 training images.

The compressed MAFusionNet-T-Lite (8.7M parameters, derived from MAFusionNet-T via structured channel pruning at ratio 0.4 and INT8 post-training quantization) achieves 89.3% mAP^50^ at 18.4 FPS on Jetson Nano with only 8.3W power consumption, significantly outperforming YOLOv13-L in throughput (18.4 vs. 8.2 FPS, 2.24× speedup) while maintaining competitive accuracy (+1.0% over YOLOv13-L-INT8 on Nano) and lower power consumption (8.3W vs. 8.6W). On Xavier NX, MAFusionNet-T achieves 41.5 FPS (1.31× over YOLOv13-L) while MAFusionNet-B achieves higher accuracy (93.8% mAP^50^) with comparable efficiency. On AGX Xavier, MAFusionNet-B achieves 94.7% mAP^50^ at 57.2 FPS with 25.8W power, approaching GPU performance with an order-of-magnitude power reduction. These results demonstrate practical feasibility for real-world edge deployment: MAFusionNet-T-Lite meets the 15+ FPS real-time processing threshold on Jetson Nano, enabling deployment in battery-powered agricultural drones or handheld scanners. The power consumption of 8.3W is within the operational budget of typical agricultural edge devices (8–12W sustained), confirming viability for field deployment scenarios.

### 5.9 Error analysis and per-class performance

To provide fine-grained understanding of model limitations, we conducted detailed error analysis. Confusion matrix analysis on the 40-class PD40 test set reveals characteristic error patterns. Most frequent inter-class confusions occur within crop-specific disease groups: Tomato Early Blight Leaf and Tomato Septoria Spot Leaf (8.2% confusion rate), Peanut Early Leaf Spot and Peanut Late Leaf Spot (6.7%), and Rice Blast and Rice Brown Spot (5.4%). These confusions occur between diseases sharing similar visual characteristics—both early blight and septoria spot exhibit irregular brown spots on tomato leaves, though early blight shows concentric ring patterns while septoria presents smaller uniformly-distributed spots. Intra-class variation contributes to these errors, as severe early-stage early blight may visually overlap with moderate-severity septoria.

Per-class AP analysis identifies categories with consistently high performance and those requiring improvement. Diseases with distinctive visual signatures achieve the highest AP scores: Corn Common Rust (AP^50^: 98.3%), Rice Blast (AP^50^: 97.8%), Potato Late Blight (AP^50^: 97.4%), and Wheat Yellow Rust (AP^50^: 97.1%). In contrast, diseases with high visual similarity to other categories show lower AP: Tomato Septoria Spot (AP^50^: 91.3%), Peanut Late Leaf Spot (AP^50^: 90.8%), and Rubber Tree Powdery Mildew (AP^50^: 90.2%). The rubber tree powdery mildew confusion arises from visual similarities with fungal deposits from other pathogens and environmental pollen under certain lighting conditions. These observations provide actionable directions for improvement: data augmentation targeting confused class pairs, curriculum learning with progressive difficulty, and specialized loss functions penalizing high-confusion pairs.

Visually Similar Disease Confusion—A Key Practical Limitation: The inter-class confusion between visually similar diseases represents a fundamental challenge that our current framework partially but not fully resolves. While MAFusionNet’s hybrid architecture reduces confusion rates compared to baselines (e.g., Tomato Early Blight vs. Septoria Spot confusion drops from 12.4% with YOLOv13-L to 8.2% with MAFusionNet-B), residual confusion persists because these disease pairs share overlapping low-level visual features (brown coloration, circular spot morphology) that even discriminative hybrid features cannot fully disambiguate without additional information. We identify three promising directions to further address this limitation: (1) *metric learning with hard-pair mining*: explicitly enforcing large embedding distances between confused disease pairs during training; (2) *multi-modal fusion*: incorporating near-infrared or hyperspectral imaging data that captures biochemical differences invisible in RGB; (3) *hierarchical classification*: first classifying the crop type and broad symptom category, then performing fine-grained disease discrimination within each group. We plan to explore these directions in future work.

False positive analysis reveals that background texture elements (soil granularity, leaf surface wax deposits, water droplets) occasionally trigger false detections for diseases with small localized symptoms. The false positive rate of 6.2% (averaged across classes) is concentrated in five disease categories representing 73% of all false positives: Peanut Nutrition Deficiency, Strawberry Powdery Mildew Leaf, Tomato Spider Mites Leaf, Rubber Tree Yellow Spot, and Wheat Smut. These false positives primarily occur when non-diseased regions exhibit superficial similarity to small lesions. Future work could incorporate negative mining strategies and attention-based background suppression to reduce these errors.

False negative analysis shows 4.8% miss rate, predominantly for very small lesions (bounding box area *<* 32^2^ pixels, corresponding to early-stage infections), severe occlusion cases, and atypical disease manifestations. Small lesion detection remains challenging despite multi-scale feature extraction, suggesting that higher-resolution feature maps or specialized small-object detection heads could further improve sensitivity for early-stage disease detection.

## 6 Conclusion

This paper presented MAFusionNet, a novel hybrid vision detection framework for plant disease identification that integrates Mamba state-space models and Transformer self-attention mechanisms through disease-aware component design. The key innovation is the explicit motivation of each architectural component by specific plant disease challenges: the MAFusion Mixer’s parallel fusion addresses the simultaneous need for sequential lesion boundary modeling and global disease-context reasoning; the SS2D-LS Block with Local-Selective enhancement preserves 2D lesion morphology while enabling efficient linear-complexity long-range modeling; and the PConv operator’s cross-shaped receptive fields capture anisotropic disease patterns such as vein-aligned blights and directional rust streaks that isotropic convolutions cannot efficiently represent.

Comprehensive experiments on the PD40 dataset demonstrated that MAFusionNet-B achieves 94.7% mAP^50^ and 81.8% mAP^50:95^, surpassing 25 state-of-the-art baselines including recent hybrid Mamba-Transformer detectors (CropMamba, HybridMamba, Mamba-DETR) by substantial margins. Ablation studies confirmed each component’s non-redundant contribution through both additive and removal experiments, with synergistic interaction effects validating the complementary design. Beyond detection accuracy, MAFusionNet demonstrates strong cross-dataset generalization, superior robustness to illumination and weather variations, and practical edge deployment feasibility: the compressed MAFusionNet-T-Lite achieves 89.3% mAP^50^ at 18.4 FPS on Jetson Nano with 8.3W power consumption, meeting real-time requirements for field-deployed agricultural monitoring systems.

The PD40 dataset, comprising 80,369 expert-verified annotated images across 40 disease categories spanning eight major crops with rigorous quality assurance (Cohen’s *κ* = 0.874 inter-annotator agreement), represents a valuable contribution to the plant disease detection research community. The dataset’s geographic diversity, multi-device acquisition, and multi-climate coverage ensure representativeness for practical agricultural deployment.

Despite these advances, several limitations and future directions merit attention. The current framework assumes single-frame detection; integrating temporal information from sequential crop monitoring images could further improve early-stage disease detection. The error analysis revealed persistent challenges with visually similar disease pairs (e.g., early blight vs. septoria spot); future work could incorporate metric learning or contrastive learning to enhance inter-class discrimination. Small lesion detection (bounding box area *<* 32^2^ pixels) remains challenging and could benefit from specialized high-resolution detection branches or super-resolution preprocessing. Finally, extending MAFusionNet to panoptic segmentation would enable pixel-level disease region delineation, providing richer information for treatment planning and precision application of agrochemicals.

The integration of efficient Mamba architectures with domain-specific Transformer attention represents a promising research direction for precision agriculture applications beyond disease detection, including nutrient deficiency assessment, yield prediction from field imagery, and integrated pest management systems. We expect MAFusionNet’s disease-aware design principles to inspire broader architectural innovations that ground hybrid model design in domain-specific visual challenges rather than generic architectural combination strategies.

## Data Availability

The datasets presented in this study can be found in online repositories. The names of the repository/repositories and accession number(s) can be found in the article/supplementary material.

## Glossary

CNN: Convolutional Neural Network
SSM: State Space Model
ViT: Vision Transformer
DETR: DEtection TRansformer
FPN: Feature Pyramid Network
mAP: mean Average Precision
IoU: Intersection over Union
FPS: Frames Per Second
PConv: Partial Convolution
CS-Mamba: Cross-Scale Mamba
SS2D-LS: Selective Scan 2D with Local-Selective enhancement
MAFusion: Multi-scale Attention Fusion
GradCAM: Gradient-weighted Class Activation Mapping
FLOPs: Floating Point Operations Per Second
EMA: Exponential Moving Average
AMP: Automatic Mixed Precision
INT8: 8-bit Integer quantization
FP16: 16-bit Floating Point precision
TensorRT: NVIDIA TensorRT inference optimization library
Model: RTX 3090YOLOv13-L, 92.8%/86.3 FPS
RT-DETRv3-L: 91.4%/71.5 FPS
CropMamba-B: 92.1%/69.2 FPS
MAFusionNet-T: 90.2%/105.8 FPS
MAFusionNet-S: 92.6%/82.4 FPS
MAFusionNet-B: 94.7%/68.5 FPS.
Model: PlatformYOLOv13-L, Jetson Nano
RT-DETRv3-L: Jetson Nano
MAFusionNet-T-Lite: Jetson Nano
YOLOv13-L: Xavier NX
RT-DETRv3-L: Xavier NX
MAFusionNet-T: Xavier NX
MAFusionNet-B: Xavier NX
YOLOv13-L: AGX Xavier
RT-DETRv3-L: AGX Xavier
MAFusionNet-T: AGX Xavier
MAFusionNet-B: AGX Xavier.
